# Pre-T cell receptor localization and trafficking are independent of its signaling

**DOI:** 10.1083/jcb.202212106

**Published:** 2023-07-26

**Authors:** Andrei I. Smid, Sam J. Garforth, Maryam S. Obaid, Hannah R. Bollons, John R. James

**Affiliations:** 1 Molecular Immunity Unit, Department of Medicine, Medical Research Council–Laboratory of Molecular Biology, University of Cambridge, Cambridge, UK; 2 Division of Biomedical Sciences, Warwick Medical School, University of Warwick, Coventry, UK

## Abstract

Expression of the pre-T cell receptor (preTCR) is an important checkpoint during the development of T cells, an essential cell type of our adaptive immune system. The preTCR complex is only transiently expressed and rapidly internalized in developing T cells and is thought to signal in a ligand-independent manner. However, identifying a mechanistic basis for these unique features of the preTCR compared with the final TCR complex has been confounded by the concomitant signaling that is normally present. Thus, we have reconstituted preTCR expression in non-immune cells to uncouple receptor trafficking dynamics from its associated signaling. We find that all the defining features of the preTCR are intrinsic properties of the receptor itself, driven by exposure of an extracellular hydrophobic region, and are not the consequence of receptor activation. Finally, we show that transitory preTCR cell surface expression can sustain tonic signaling in the absence of ligand binding, suggesting how the preTCR can nonetheless drive αβTCR lineage commitment.

## Teaser

Smid, Garforth, et al. reconstitute pre-T cell receptor (preTCR) expression in non-immune cells to show that signaling is not required for its rapid internalization and degradation observed during T-cell development, and that the preTCR can drive tonic signaling in T cells.

## Introduction

T cells are an essential white blood cell–type of the immune systems of many animals, including our own. They are responsible for both the detection and elimination of many pathogens, such as bacteria, viruses, as well as some cancerous cells. The specificity and function of T cells are instructed during their development in the thymus, primarily during childhood. During this period, immature T cells (known as thymocytes) must pass through several checkpoints during their development in the thymus to ensure they create a functional T cell antigen receptor (TCR) complex that does not recognize peptide ligands derived from host proteins (Germain, 2002; Hayday and Pennington, 2007). One of the first of these checkpoints, during the double negative 3 (DN3) stage, is the selection of thymocytes that have productively rearranged the TCRβ gene in a process known as β-selection (Hayday and Pennington, 2007). Thymocytes are rescued from apoptosis at this checkpoint by transiently expressing the pre-T cell antigen receptor (preTCR), a complex of the nascently expressed TCRβ chain, an invariant pTa chain, and accessory CD3 signaling proteins (Dutta et al., 2021). The unique pTa chain includes an extensive intracellular sequence that appears largely unstructured and is not well conserved across species but may have a role in preTCR function (von Boehmer, 2005). Signaling from the preTCR induces proliferation, inhibits further recombination at the TCRβ locus, and initiates commitment to the αβ-T cell lineage. However, concomitant successful rearrangement of the γδTCR leads to signaling that instead biases thymocytes toward the γδ-T cell lineage. This same bias toward the γδ-T cell lineage appears if the complete αβTCR is prematurely expressed by transgenic modification (Borowski et al., 2004).

How though can thymocytes at the β-selection checkpoint differentiate between signals from the preTCR and γδTCR/αβTCR receptors, given they are structurally similar and signal through ITAM motifs on the same set of accessory CD3 chains? Experiments with transgenic mice favor a TCR “signal strength” model, which proposes that “stronger” TCR signaling from the γδTCR promotes uncommitted thymocytes to adopt the γδ-T cell fate whilst “weaker” signaling from the preTCR promotes the αβ-T cell fate (Zarin et al., 2014; Hayes et al., 2005; Haks et al., 2005). The preTCR is generally thought to signal in a ligand-independent manner and produce quantitively lower signaling compared with γδTCR (Irving et al., 1998; Dutta et al., 2021; Gibbons et al., 2001), though signal strength is rarely defined.

Surface expression of the preTCR is typically 100-fold lower than the TCR on T cells (Borst et al., 1996), and when at the cell surface, the receptor appears to undergo constitutive internalization and degradation in the lysosomes in the absence of a ligand (Panigada et al., 2002; Groettrup et al., 1993; Yamasaki et al., 2006; Wilson et al., 1995; Maguire et al., 1990; Kearse et al., 1994). Although barely detectable at the plasma membrane at steady state, the translocation of the preTCR to the plasma membrane is required as ER-retained preTCR complexes are unable to rescue the development of thymocytes arrested at the double-negative stage (O’Shea et al., 1997). The requirement for cell surface expression is further supported by the fact that the activity of LCK, the kinase that initiates T cell activation, is required for β-selection in thymocyte development (Wallace et al., 1995; Molina et al., 1992). Pertinently, LCK is a plasma membrane–bound kinase, therefore underlining the importance of preTCR localization to this compartment (Marchildon et al., 1984). The low surface expression and lysosomal degradation of the preTCR have invariably been explained as a consequence of ligand-independent signaling of the receptor, leading to efficient internalization. However, despite a range of structural studies and genetic experiments in mice, many aspects of the biochemical and signaling properties of the preTCR are still debated.

Elucidating preTCR function is necessary both for a fundamental understanding of immune cell biology and also for how it goes wrong, causing significant deleterious effects on a person’s immune system. There is also clinical relevance since recent attempts to use induced pluripotent stem cells as “off-the-shelf” CAR-T cancer therapies require knowledge of how thymocytes cross the β-selection checkpoint appropriately and develop along the αβ-T cell pathway (van der Stegen et al., 2022). Unfortunately, studying the molecular details of preTCR function is hindered by the temporary nature of its expression, which is found to be at very low levels at the cell surface and only during a small period in the DN3 stage of thymocyte development. Furthermore, the small number of DN3 thymocytes are difficult to extract from living tissue and these primary cells are not readily amenable to transfections or genetic manipulations. As thymocytes express signaling kinases, it is also difficult to distinguish the intrinsic localization of the receptor from the consequences of receptor signaling.

To overcome these challenges, we have reconstituted expression of the complete preTCR complex in non-immune cells so that the fundamental biology of the receptor can be explored in a far more tractable manner, as we have previously done for the TCR (James and Vale, 2012). This enabled us to compare the preTCR and αβTCR receptors directly and to study their localization and trafficking with a much greater range of techniques. We find that the distinguishing features of the preTCR, namely its low surface expression, rapid internalization from the plasma membrane, and efficient lysosomal targeting, are all intrinsic properties of the preTCR itself; signaling is not required. We also show that the preTCR is a monovalent receptor. Finally, we provide evidence that, like the αβTCR, the preTCR can signal tonically in the absence of ligand binding, which could allow its surface expression alone to drive β-selection.

## Results

### Reconstituted preTCR expression shows poor surface localization

**Figure 1.**
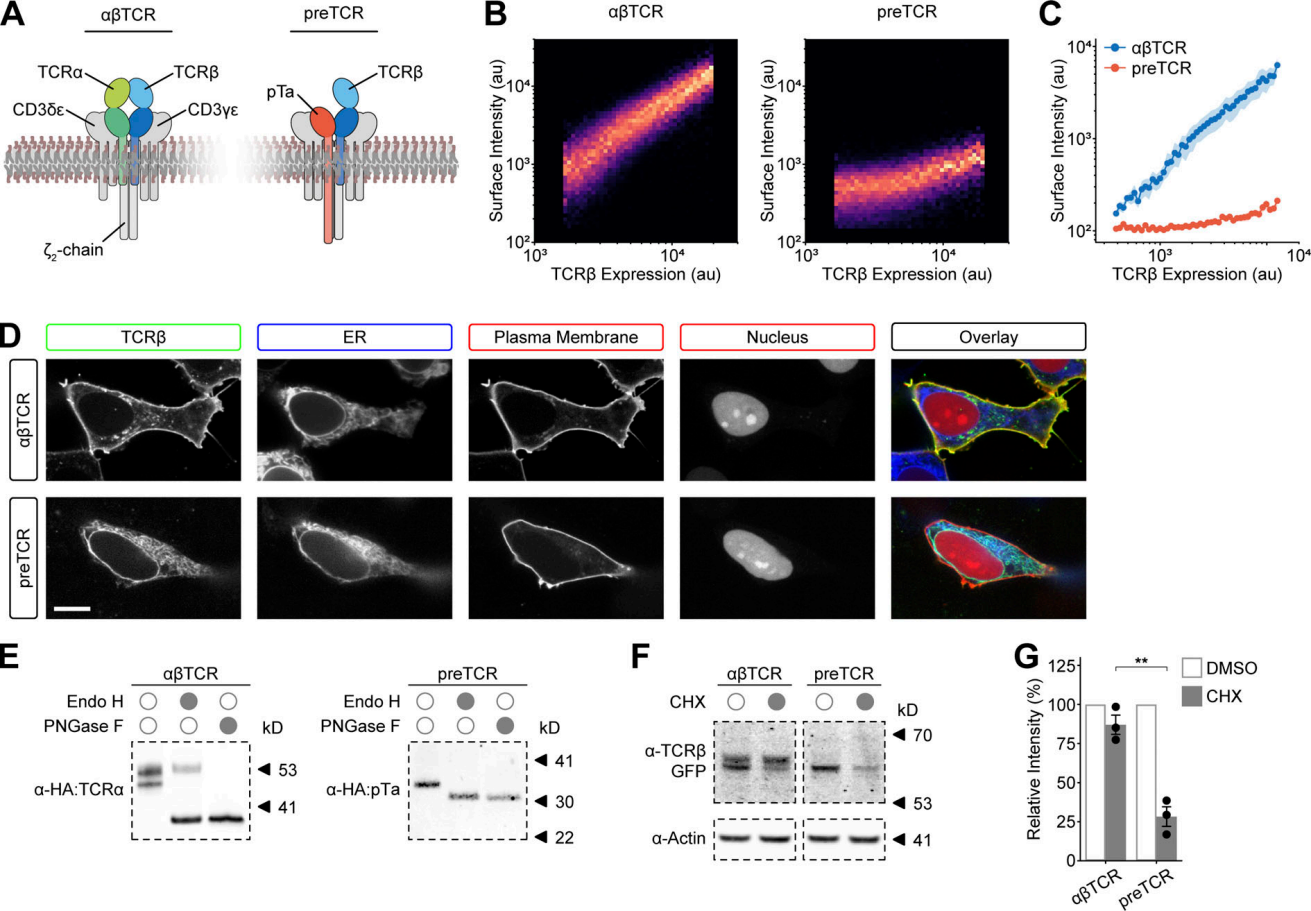
**ER localization of the preTCR complex in reconstituted HEK cells. (A)** Schematic showing the constituent protein chains of the αβTCR and preTCR complexes within the plasma membrane. **(B)** HEK cells were transfected with constructs to express the complete αβTCR or preTCR complexes. Raw flow cytometry plots show the correlation between receptor expression (measured by TCR β-GFP) and surface staining (anti-TCRβ-AF647). **(C)** Quantification of multiple datasets derived from B, where bounding area around data points shows mean ± SEM (*n* = 3), with P < 0.01 at all expression levels. **(D)** HEK cells were cotransfected with constructs for αβTCR or preTCR (GFP fused to TCRβ), along with ER-localized BFP, plasma membrane–localized mCherry, and nuclear-localized iRFP fluorophores. Representative images are shown, and colored boxes denote protein representation in the overlay image. Scale bar, 10 μm. **(E)** HEK cells expressing the αβTCR or preTCR were lysed and the complexes purified through the GFP fused to TCRβ chains. Isolated complexes were incubated with either EndoH or PNGaseF enzymes to remove defined glycosylation moieties. Samples were then subjected to Western blot analysis, probing for the HA epitope expressed at the cleaved N-terminus of TCR α or pTa. The blot shown is representative of three replicates. **(F)** HEK cells expressing the denoted receptor were treated with CHX overnight or vehicle control (DMSO) before purifying TCRβ as in A. Samples were blotted for GFP (fused to TCRβ) and actin as a loading control. **(G)** Quantification of Western blots as in F, with integrated band density for each receptor normalized to control. Datapoints show mean ± SEM (*n* = 3); asterisks indicate P < 0.01 when comparing αβTCR and preTCR datasets in the presence of CHX. A two-tailed, two-sided *t* test was used for all statistical analyses. Source data are available for this figure: SourceData F1.

We have previously reconstituted the complete TCR complex in HEK293T cells (hereafter, HEK cells), which are a non-immune cell line (James and Vale, 2012; Liaunardy-Jopeace et al., 2017). Given this success, we wanted to investigate whether the preTCR complex could also form in the absence of T cell–specific factors (Fig. 1 A). We therefore cloned the human *PTCRA* gene in place of the *TCRA* equivalent in our original constructs. The *TCRB* sequence was derived from the G10 TCR clonotype (V_β_5.1) for which a TCRβ-specific antibody exists (clone LC4). To provide the most direct control for preTCR expression, we also created constructs of the complete G10 TCR complex using its *TCRA* sequence (V_α_28.1). As expected from our previous work, the G10 αβTCR complex (hereafter, αβTCR) was well expressed in transiently transfected HEK cells, as measured by fluorescence from GFP fused to TCRβ, and showed highly correlated cell surface staining using a fluorophore-conjugated anti-TCR V_β_5.1 antibody (Fig. 1, B and C). However, transient expression of the preTCR complex (pTa, TCRβ, CD3γδε, and ζ-chain) was barely detectable at the cell surface and only at very high expression levels (Fig. 1, B and C). Given the structural similarities between the two complexes, we were keen to investigate why there was such a difference in relative surface expression.

### preTCR complexes do not readily form in the ER

Our initial hypothesis to explain the low-surface expression of the preTCR was impaired receptor complex formation within the ER of HEK cells. To investigate the potential retention of the preTCR within the ER, we used confocal microscopy to directly localize the receptor complexes. When expressed as part of the preTCR complex, the GFP-tagged TCRβ chain was almost exclusively localized to the ER (Fig. 1 D), whereas robust cell surface localization was observed when TCRβ was expressed within the αβTCR complex (Fig. 1 D), supporting the idea that the majority of the preTCR was retained in the ER.

Transmembrane proteins are translated and folded within the ER; during this initial folding, a branched glycan moiety is often transferred to specific asparagine residues within the nascent polypeptide chain. The successful egress of a protein from the ER allows the extensive modification of this glycan structure within the Golgi compartment before it is trafficked to the plasma membrane. The presence of more complex glycans on a protein can thus be used to detect whether a protein has been trafficked from the ER or not. A common means to do this is determining what fraction of the glycoprotein is sensitive to endoglycosidase H (EndoH) since this enzyme is incapable of cleaving the glycan moiety once significant processing has occurred in the Golgi compartment. We therefore used EndoH sensitivity as an additional measure of preTCR ER retention.

Isolating the GFP-tagged αβTCR complex from transfected HEK cells and detecting HA epitope-tagged TCRα showed two bands by Western blotting, indicating differential glycosylation of the TCRα subunit (Fig. 1 E). EndoH treatment of this purified fraction caused a decrease in the molecular weight of the lower band but a significant fraction of the higher molecular weight band was EndoH resistant (Fig. 1 E). To confirm that this resistant band was due to diversified glycosylation, we used PNGase F to remove all sugar moieties, which eliminated this fraction (Fig. 1 E). The equivalent treatment of HEK cells expressing the preTCR had only one detectable band for the purified HA-tagged pTa subunit, which was completely sensitive to EndoH (Fig. 1 E). This suggested that the bulk of the pTa chain, and hence the preTCR complex, was retained in the ER rather than trafficked to the Golgi compartment.

Sustained localization of the preTCR subunits within the ER exposes the proteins not bound within a complex to increased protein degradation through the ER-associated protein degradation (ERAD) pathway. We blocked new protein synthesis in HEK cells expressing the preTCR or αβTCR using cycloheximide (CHX) so that we could measure the lifetime of the TCRβ chain within each complex. CHX treatment caused a small decrease in TCRβ protein levels when expressed as part of the αβTCR (Fig. 1, F and G). Pertinently, it was primarily the lower molecular weight band that decreased in intensity (Fig. 1 F), implying that the exit of the αβTCR complex from the ER protected it from degradation. Conversely, the TCRβ subunit of the preTCR was very susceptible to protein degradation (Fig. 1, F and G), with a loss of 75% total protein (Fig. 1 G). These results emphasize that the chains of the preTCR reside predominantly within the ER rather than later compartments of the trafficking pathway.

### Surface-localized preTCR complexes are readily detectable

**Figure 2.**
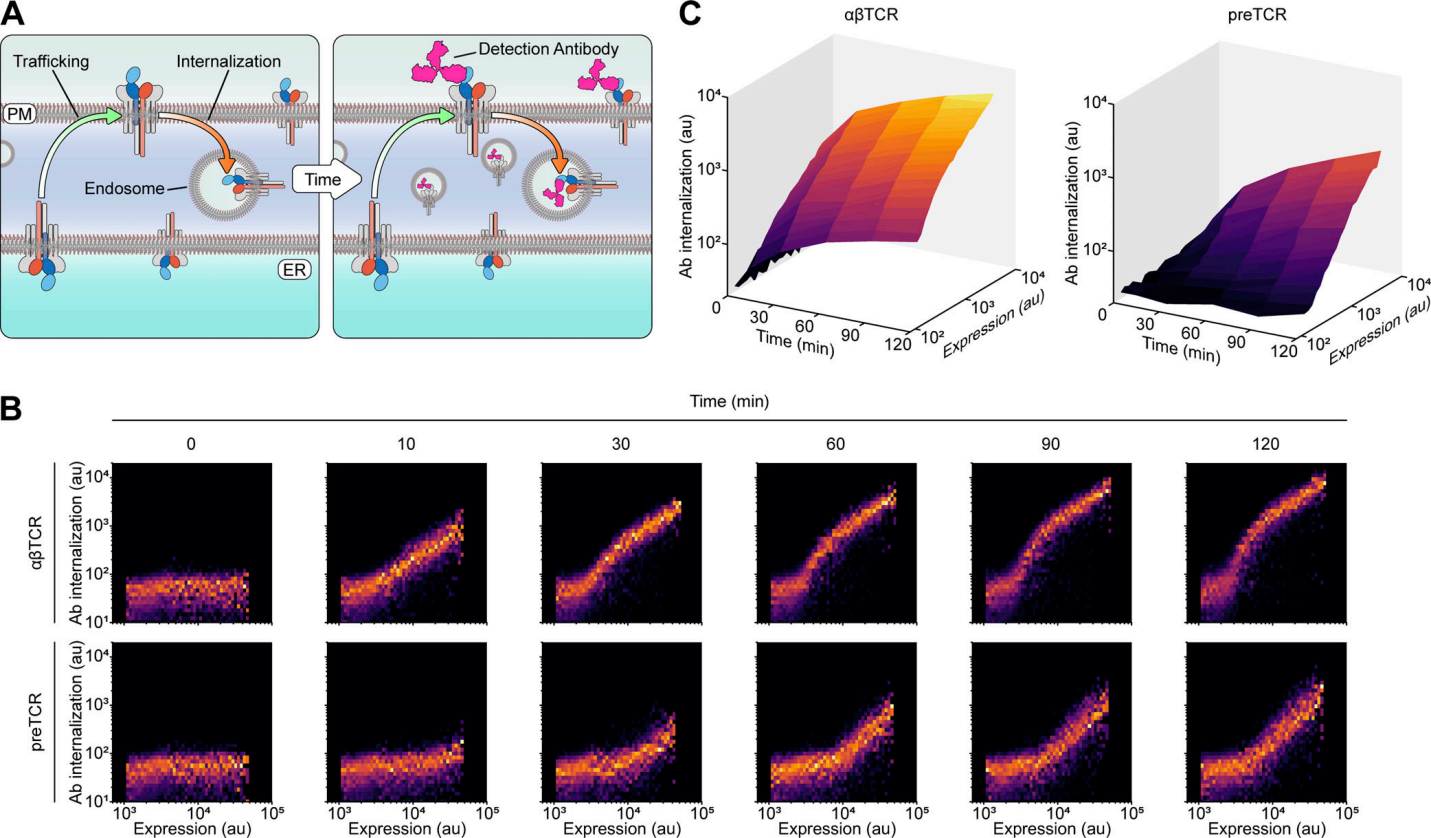
**preTCR complexes can traffic to the cell surface in HEK cells. (A)** Schematic describing the internalization assay, where antibody binding to cell surface receptors can be quantified through the time-dependent increase in cell fluorescence when labeled antibodies become internalized. PM, plasma membrane; ER, endoplasmic reticulum. **(B)** Raw flow cytometry plots of one repeat of the internalization assay applied to αβTCR and preTCR-expressing HEK cells. The time-dependent increase in fluorescence intensity is plotted against receptor expression (quantified by TCRβ-GFP). **(C)** Quantification of internalization assay with cellular fluorescence plotted as a function of both time and receptor expression. Dataset is representative of two biological replicates.

**Figure S1.**
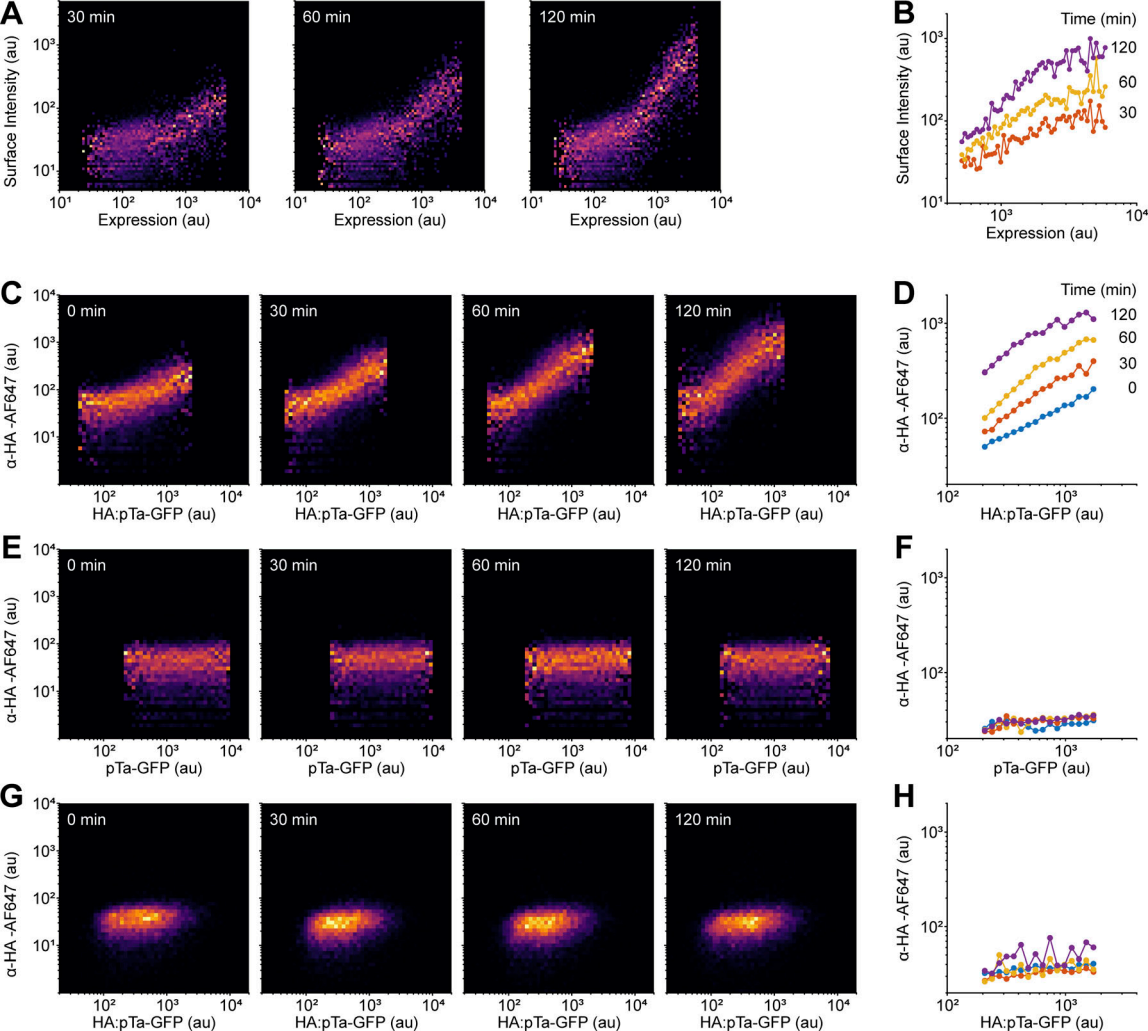
**preTCR internalization in HeLa and Jurkat T cells. (A)** HeLa cells were transfected with vectors encoding the preTCR complex, similarly as for HEK cells in the main text. Transfected HeLa cells were then incubated with APC-conjugated anti-V_β_5.1 antibody for the indicated time at 37°C before detachment from well and fixation. Raw flow cytometry plots are shown for one representative experiment from two replicates. **(B)** Quantification of the mean surface staining from A at each time point, as a function of expression level. **(C)** Jurkat T cells were lentivirally transduced with the HA-tagged pTa gene sequence, which was also fused to GFP for facile detection of expression levels. Transduced Jurkat cells were incubated with AF647-conjugated anti-HA antibody for indicated time at 37°C before washing and cell fixation. Raw flow cytometry plots are shown for one representative experiment from two replicates. **(D)** Quantification of the mean surface staining from C at each timepoint, as a function of expression level. **(E)** Jurkat T cells were equivalently transduced and assayed as in C but with a pTa sequence without the HA epitope, to confirm that antibody internalization was HA-specific. Raw flow cytometry plots are shown for one representative experiment from two replicates. **(F)** Quantification of the mean surface staining from E at each time point, as a function of expression level. **(G)** The *TCRB*-negative Jurkat line (J.RT3) was transduced with the HA-tagged pTa gene sequence and assayed as in C. No antibody was detectable in the absence of TCRβ expression, showing that preTCR internalization in Jurkats required full complex formation. Raw flow cytometry plots are shown for one representative experiment from two replicates. **(H)** Quantification of the mean surface staining from G at each time point, as a function of expression level.

**Figure S2.**
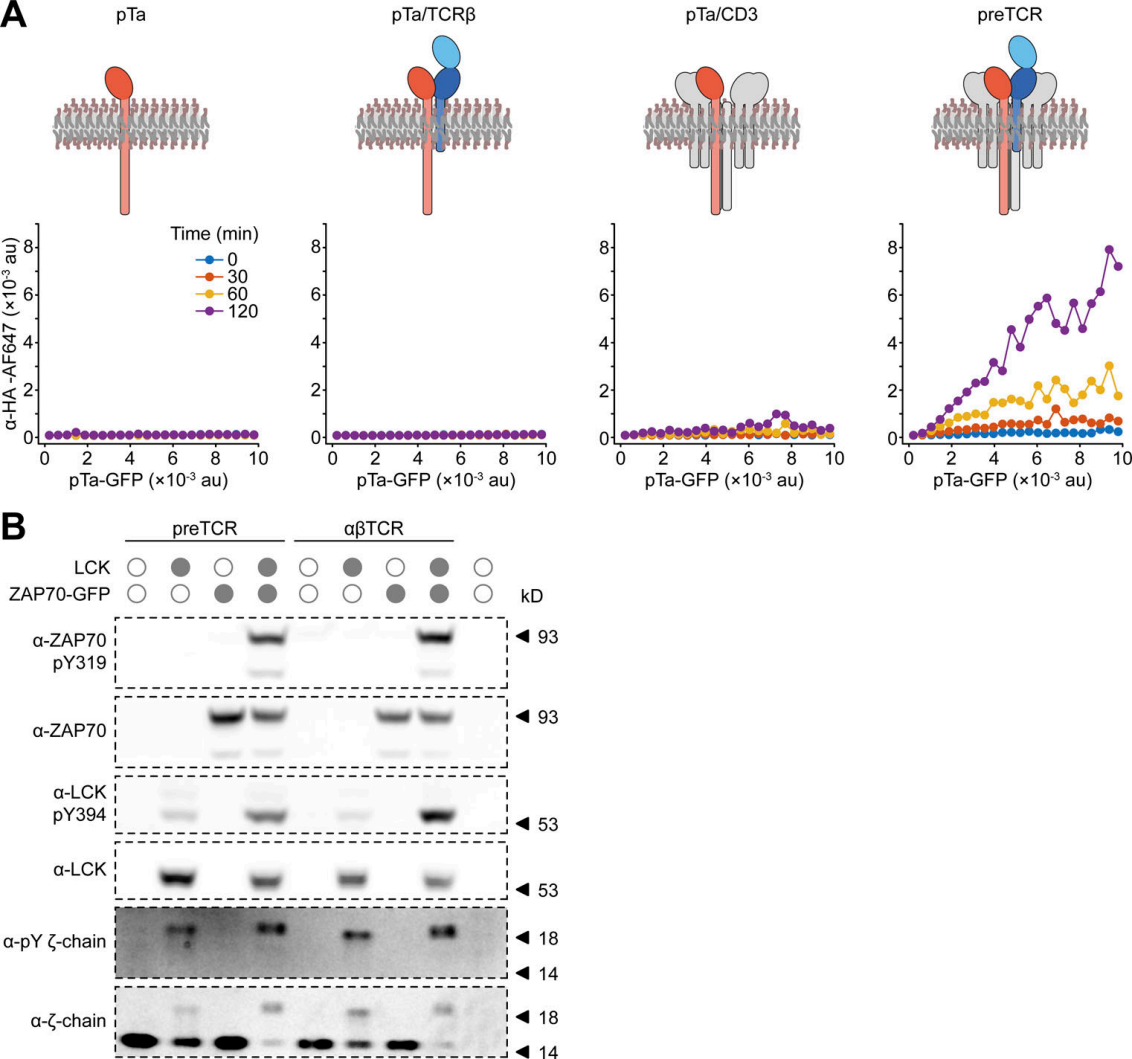
**preTCR internalization requires all complex components and is not constitutively phosphorylated in HEK cells. (A)** The internalization assay was applied to HEK cells expressing HA-tagged pTa protein chain in the presence of defined components of the preTCR complex. An AF647-conjugated anti-HA antibody was used to quantify potential pTa internalization. Schematics above each plot show the chains expressed in HEK cells. Only in the presence of all preTCR components can the anti-HA antibody become internalized. One representative experiment from two replicates is shown. **(B)** HEK cells expressing the preTCR complex or additionally with LCK or ZAP70 kinases as denoted, were lysed and subjected to Western analysis. Blotting with the indicated antibodies shows that only in the presence of exogenously expressed kinases is CD3 ζ-chain phosphorylation detectable. One representative experiment from two replicates is shown. Source data are available for this figure: SourceData FS2.

Constitutive retention of the preTCR complex within the ER would readily explain the low surface expression in HEK cells. To confirm that the preTCR did not exit the ER, we incubated HEK cells expressing the preTCR complex at 37°C with a fluorescently labeled TCRβ-specific antibody for defined periods (Fig. 2 A). If the preTCR were in fact capable of trafficking to the cell surface, it could bind the labeled antibody before the receptor was internalized, leading to the gradual accumulation of intracellular fluorescence; this would not be the case for ER-retained preTCR complexes (Fig. 2 A). Contrary to our expectations, we found strong evidence for preTCR cell surface expression, with HEK cells expressing the preTCR becoming substantially more fluorescent over time (Fig. 2 B). The αβTCR complex, by comparison, stained rapidly after only a few minutes, indicative of its higher residence at the cell surface (Fig. 2 B). Quantifying these datasets with respect to receptor expression showed that the internalization of the preTCR could be detected even at the lowest levels (Fig. 2 C). We also observed the same internalization effect for the preTCR complex in HeLa and Jurkat T cells (Fig. S1). We also confirmed that it was the complete preTCR complex trafficking to the cell surface (Fig. S2 A).

These results show that the preTCR complex can indeed form intrinsically in non-thymocytes and is able to transit to the cell surface, but its steady state surface expression is very low compared to the αβTCR complex. This phenotype is strikingly similar to that found in developing thymocytes but without any signaling that would normally be concomitantly overlaid. To make certain that the preTCR was not stimulated when expressed in HEK cells, we probed for phosphorylation of the preTCR ζ-chain and as expected, could not detect any modification without addition of exogenous kinases (Fig. S2 B).

### Rate of preTCR trafficking to the cell surface is equivalent to αβTCR

**Figure 3.**
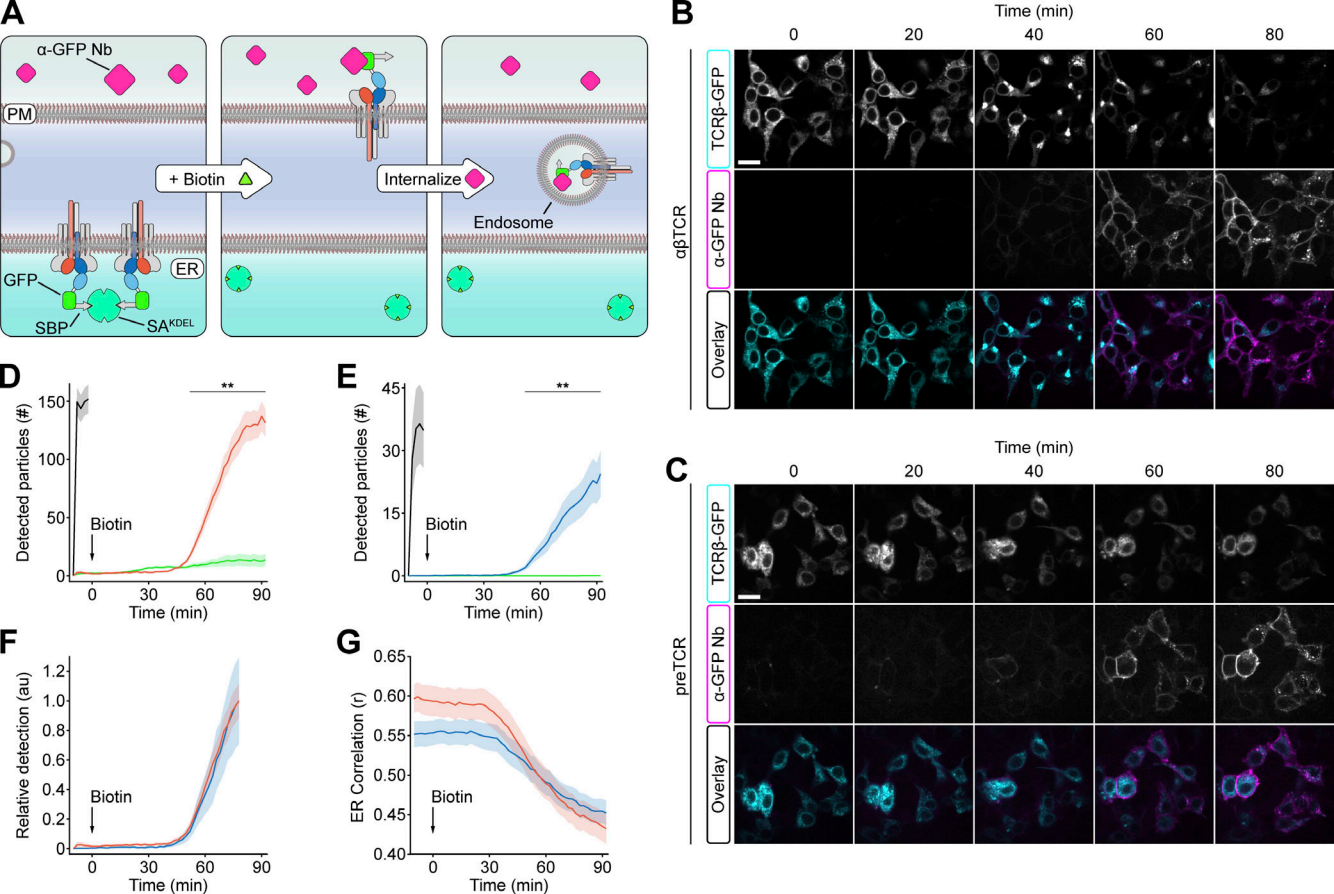
**Equivalent ER export is observed for the αβTCR and preTCR complexes. (A)** Schematic showing RUSH assay applied to preTCR ER export. SBP-GFP fused to the cleaved N-terminus of TCRβ causes ER retention through binding to streptavidin (SA^KDEL^). Biotin addition causes the release of this lock and synchronous export of complexes from ER, where the receptors can bind extracellular anti-GFP nanobody (Nb) and internalize it into endosomes. **(B)** Selected images showing the binding and internalization of anti-GFP Nb when SBP–αβTCR complexes are released from ER on biotin addition. Colored boxes denote protein representation in the overlay image. Scale bar, 20 μm. **(C)** Equivalent images as in B but for the preTCR complex when released from the ER. **(D)** Quantification of vesicles containing internalized anti-GFP Nb when SBP–αβTCR is released on biotin addition (red). Equivalent data for αβTCR without SBP (black) is shown, highlighting the rate of Nb internalization without retention. Control data for SBP–αβTCR without biotin is also presented (green). Bounding area around datapoints shows mean ± SEM of multiple fields of view from six biological replicates; asterisks indicate P < 0.01 when comparing αβTCR datasets with biotin addition or not. **(E)** Equivalent quantification as in D for the SBP-preTCR (blue) RUSH assay. **(F)** Biotin addition plots from D and E normalized to detected particles at 75 min. **(G)** The correlation coefficient between the ER marker and receptor image channels with time is plotted for both αβTCR (red) and preTCR (blue) biotin addition datasets. The bounding area around data points shows mean ± SEM of multiple fields of view from six biological replicates. A two-tailed, two-sided *t* test was used for all statistical analyses.

We next wanted to ascertain whether the preTCR was retained in the ER due to inefficient complex formation or its decreased transport from the ER to the cell surface. To elucidate which explanation is more plausible, we made use of the RUSH assay (Boncompain et al., 2012), which traps proteins within the ER lumen using the streptavidin-binding protein (SBP) interaction. Biotin addition causes the release of this trap and drives the synchronous movement of cargo through the anterograde trafficking pathway (Fig. 3 A). If preTCR complex formation is inefficient, then the RUSH assay provides sufficient time for subunits to combine together and so the synchronized exit rate of the receptors from the ER should be comparable.

To apply the RUSH assay to the preTCR, we fused the SBP peptide sequence to the N-terminus of TCRβ in combination with an ER “hook” constructed from streptavidin-KDEL (ERSA^KDEL^; Fig. 3 A). Because the preTCR was so poorly expressed at the cell surface at steady state, we used the temporal accumulation of internalized receptors as a more robust measure of receptor trafficking. To specifically measure internalized receptors, we also installed GFP at the extracellular terminus of TCRβ (after SBP) in both the preTCR and αβTCR complexes. We could then incubate HEK cells expressing the receptors with a fluorescently labeled nanobody specific to GFP (anti-GFP Nb), which cannot enter the cell unless directly bound to a receptor that has been internalized from the cell surface (Fig. 3 A).

Coexpression of the SBP-tagged receptors with the hook completely disrupted surface expression, which could be restored on biotin addition by releasing ER retention (Fig. 3, B and C). We then used an imaging assay to measure the rate of receptor trafficking from the ER to the cell surface as a correlate of the ER exit rate. HEK cells expressing the preTCR or αβTCR were first incubated with the anti-GFP Nb before biotin was added to release the lock on receptor anterograde trafficking. Within 40 min of biotin addition, receptors could be detected reaching the cell surface, as judged by the increased fluorescence from GFP nanobody surface binding and internalized vesicles (Fig. 3, B and C; and Videos 1 and 2). Quantifying the rate of this fluorescence increase with time for the two receptors showed them to be comparable (Fig. 3 F), though the steady-state values were different, as expected (Fig. 3, D and E). We also measured the correlation between the receptors and BFP-labeled ERSA^KDEL^ to estimate the initiation of egress from this compartment (Fig. 3 G). We found a decreased correlation for both the preTCR and αβTCR starting ∼30 min after biotin addition, which preceded the detection of internalized anti-GFP Nb by ∼10 min (Fig. 3 G). This assay demonstrated that, once formed, the preTCR and αβTCR have a broadly equivalent capacity to traffic to the plasma membrane and reinforces the assertion that nascent preTCR complex formation is not efficient, at least in HEK cells.

**Video 1.** **RUSH assay to measure anterograde trafficking of αβTCR complex.** Movie shows the addition of biotin (t = 0 min) to release αβTCR complexes retained in the ER using the RUSH assay. Video is related to Fig. 3 B of the main text. Of note, nanobody binding substantially decreases GFP fluorescence, hence surface-localized GFP-fused αβTCR receptors are not easily observed in the left panel. Colored boxes denote protein representation in the overlay image. Scale bar, 10 μm. Images were collected at 0.5 frames/min, with a playback rate of 10 frames/s.

**Video 2.** **RUSH assay to measure anterograde trafficking of preTCR complex.** The movie shows the addition of biotin (t = 0 min) to release preTCR complexes retained in the ER using the RUSH assay. The video is related to Fig. 3 C of the main text. Colored boxes denote protein representation in the overlay image. Scale bar, 10 μm. Images were collected at 0.5 frames/min, with a playback rate of 10 frames/s.

### The preTCR complex is rapidly removed from the cell surface

**Figure 4.**
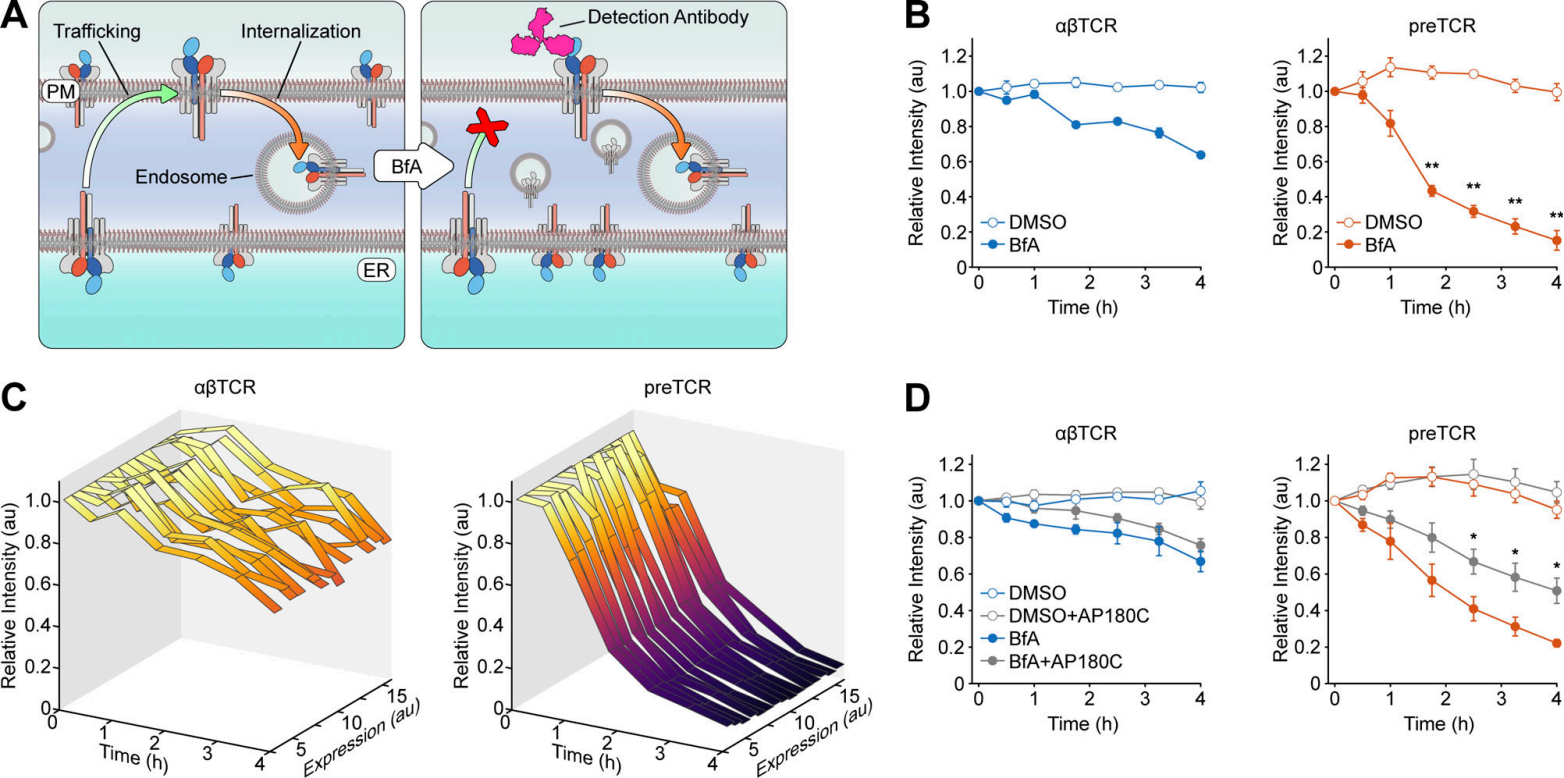
**preTCR complex is rapidly internalized from cell surface. (A)** Schematic demonstrating the brefeldin A (BfA) assay, where anterograde flow of newly formed receptors is blocked, so that the internalization rate of surface-expressed receptors can be measured in isolation. **(B)** HEK cells expressing either the αβTCR or preTCR were incubated for a defined period with either BfA or DMSO vehicle control before receptors still present at plasma membrane were detected with an anti-TCR β antibody. The decrease in intensity from the initial time point follows the sustained internalization of the receptors. Data points show mean ± SEM (*n* = 3); asterisks indicate P < 0.01 when comparing αβTCR and preTCR datasets in presence of BfA. **(C)** Data from one representative BfA assay, where the internalization rate is shown at different binned expression levels based on the range of transfection in HEK cells. **(D)** Repeat of the BfA assay as in B but also performed with HEK cells coexpressing AP180C, which disrupts clathrin-mediated endocytosis. Datapoints show mean ± SEM (*n* = 3); asterisks indicate P < 0.05 when comparing effect of AP180C on preTCR datasets in presence of BfA. A two-tailed, two-sided *t* test was used for all statistical analyses.

**Figure S3.**
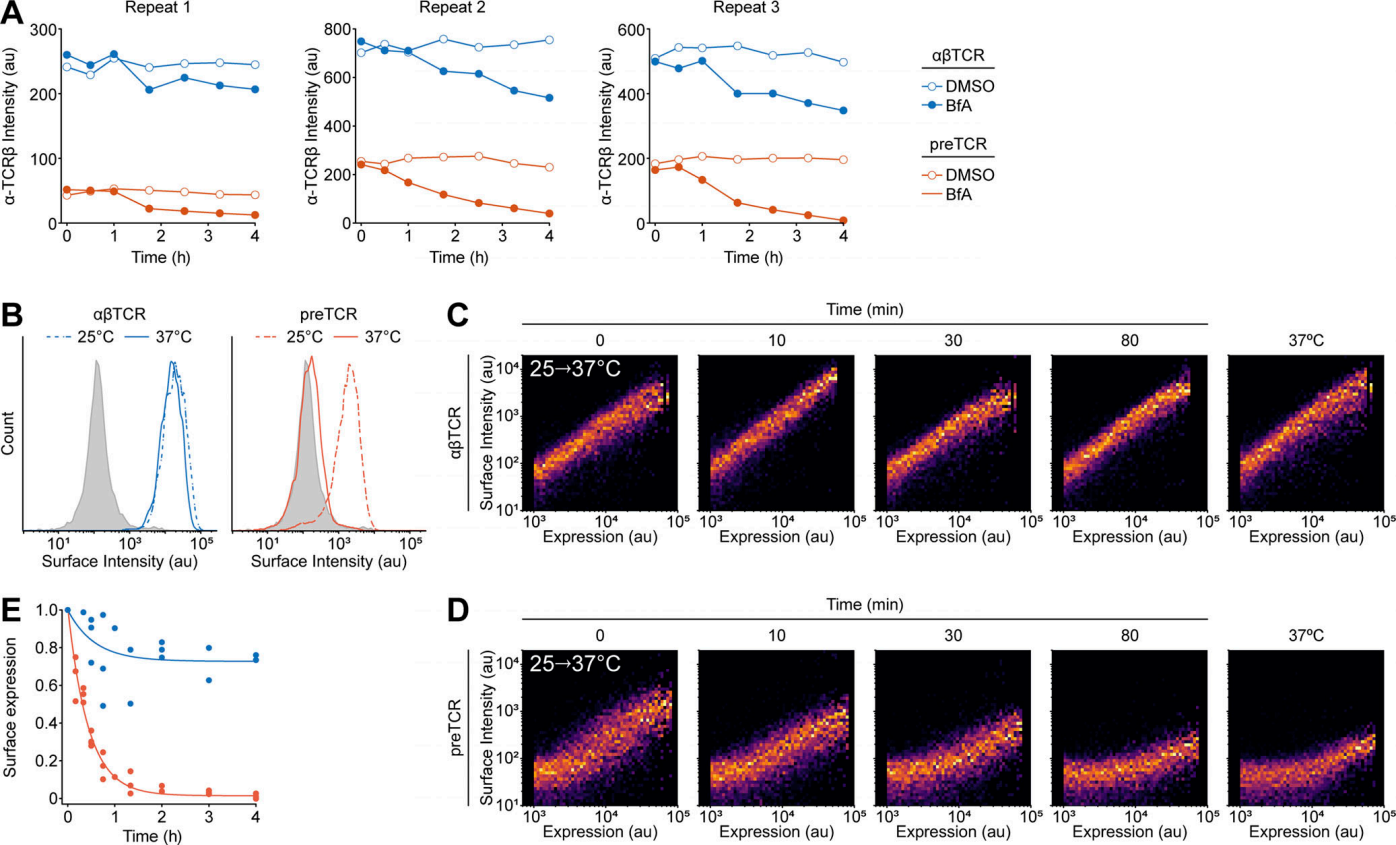
**preTCR complex is stabilized at cell surface at lower temperatures. (A)** Plots show the individual unnormalized datasets used to construct Fig. 4 B. **(B)** Flow cytometry of surface-stained HEK cells stably expressing either the αβTCR or preTCR complexes, when incubated at either 37°C (solid line) or 25°C (dashed line). Filled histogram denotes staining of untransduced HEK cells. **(C)** Flow cytometry plots showing surface staining of αβTCR as a function of expression when transfected HEK cells are rapidly transitioned from 25°C to 37°C. Indicated time denotes the period since temperature shift, with the final plot showing αβTCR-expressing HEK cells maintained at 37°C. Dataset is representative of three biological replicates. **(D)** Equivalent dataset as in B for the preTCR complex. **(E)** Relative mean surface staining of αβTCR and preTCR expressed in HEK cells as a function of time after temperature shift. Datapoints have been normalized to the initial value when cells are maintained at 37°C and are shown accumulated from three biological replicates.

Given that anterograde transport of the preTCR was comparable with the αβTCR, the low surface expression phenotype of the preTCR could be explained by some combination of poor preTCR complex formation within the ER and increased internalization from the cell surface. We therefore investigated whether the preTCR complex was more rapidly endocytosed from the cell surface. To isolate this part of the trafficking pathway, we used brefeldin A (BfA) to disrupt the trafficking of nascent preTCR complexes from the ER to the cell surface (Fig. 4 A). For the αβTCR, acute incubation with BfA caused a slow decrease in the steady-state levels of the receptor at the cell surface over 4 h (Fig. 4 B and Fig. S3 A). The low but detectable surface expression of the preTCR, already significantly decreased compared with the αβTCR, was almost entirely lost with the same BfA treatment. (Fig. 4 B and Fig. S3 A). This increased rate of endocytosis was equivalent at all levels of preTCR expression (Fig. 4 C).

Basal TCR internalization in the absence of ligand binding is thought to occur at least partially through clathrin-mediated endocytosis (CME; Charpentier and King, 2021), so we speculated that this would be the case for the preTCR in HEK cells too. Thus, we repeated the BfA assay but now coexpressed AP180C, a dominant negative form of the AP180 protein required for CME (Zhao et al., 2001). We found a significant decrease in the rate of preTCR internalization when CME was disrupted, demonstrating that this process plays a significant role in the internalization of the receptor (Fig. 4 D). We also found that the steady-state surface levels of the preTCR could be increased by culturing the cells at 25°C (Fig. S3, B–D), a phenotype that is strikingly like that reported for peptide-free MHC class-I complexes (Ljunggren et al., 1990; Montealegre et al., 2015). This increase in surface expression was quickly reversed by returning the cells to 37°C (Fig. S3 E).

**Figure S4.**
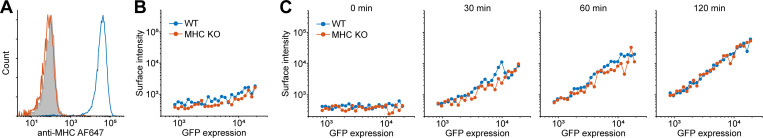
**MHC expression on HEK cells does not affect preTCR internalization. (A)** Confirmation of complete MHC-I knockout in HEK cells. Wildtype (WT) HEK cells (blue) stained with AF647-labeled anti-MHC antibody show high expression of MHC that is completely absent on MHC-knockout cells (MHC KO; red) and is indistinguishable from isotype control staining (filled grey). **(B)** preTCR expression in either WT HEK cells or MHC KO cells shows no substantial difference in surface staining with APC-conjugated anti-V _β_5.1 TCR antibody. **(C)** Repeating the preTCR internalization assay with WT and MHC KO HEK cells shows no substantial difference in the absence of MHC at any timepoint measured. One representative experiment from two replicates is shown.

A recent study has suggested that MHC proteins on stromal cells within the thymus can act as ligands for the preTCR complex, with loss of thymic MHC expression causing a deviation in DP thymocyte integrity that has passed β-selection (Duke-Cohan et al., 2022). To test whether the rapid internalization we found for the preTCR expressed in HEK cells might somehow be due to MHC class-I expression on HEK cells, we used CRISPR/Cas9 to remove its normal expression. Using MHC^KO^ HEK cells (Fig. S4 A) had no effect on the surface expression (Fig. S4 B) or internalization of the preTCR (Fig. S4 C), demonstrating that our results could not somehow be explained by MHC binding either *in cis* or *trans*.

In combination, these datasets implied that once localized at the cell surface, the preTCR complex is far more susceptible to sustained internalization (without any recycling) compared with the αβTCR.

### The preTCR complex is routed to lysosomes after internalization

**Figure 5.**
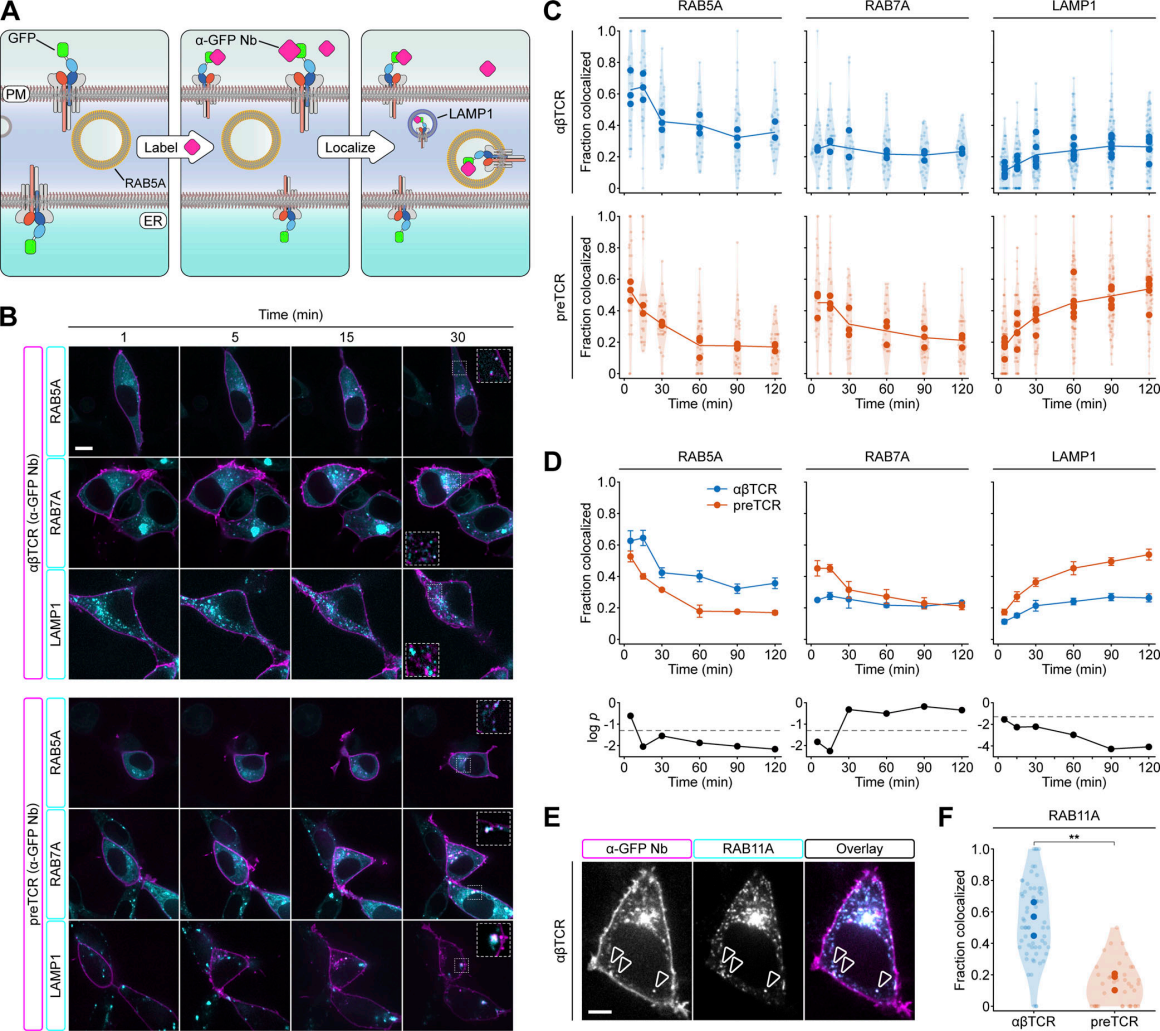
**preTCR complex is constitutively trafficked to lysosomes. (A)** Schematic describing the localization of internalized receptors to distinct parts of the endosomal pathway. GFP-fused receptors localized at the cell surface can bind anti-GFP nanobody (Nb) and internalize the fluorescently labeled Nb. Cotransfection with labeled RAB5A/RAB7A and LAMP1 constructs allows the identification of internalized receptor compartment with time. **(B)** Representative images from the colocalization assay for the αβTCR and preTCR complexes internalizing Nb with time. Colored boxes denote protein representation in the overlaid images. Scale bar, 10 μm. For all inset images, the bounding area is 8 μm in width. **(C)** Quantification of colocalization of internalized Nb-bound receptors with early (RAB5A) or late (RAB7A) endosomes and lysosomes (LAMP1). Data points show mean colocalization from three independent experiments with individual values and kernel density for the complete dataset also presented. The line shows the overall mean of combined experiments. **(D)** Datasets from C were combined to allow for direct comparison between αβTCR and preTCR complexes. Upper panels show mean ± SEM (*n* = 3 or 6) and lower panel shows *t* test statistic (P) comparing αβTCR and preTCR datasets. Dashed lines indicate P = 0.05. **(E)** Representative image showing colocalization of internalized Nb-bound αβTCR with RAB11A, a marker of recycling endosomes. Arrows mark exemplar colocalized spots and colored boxes denote protein representation in the overlaid image. Scale bar, 5 μm. **(F)** Quantification of colocalization of internalized Nb-bound receptors with recycling (RAB11A) endosomes, measured 5–20 min are Nb addition. Data points show mean colocalization from three independent experiments with individual values and kernel density for the complete dataset also presented. Asterisks indicate P < 0.01 when comparing αβTCR and preTCR datasets. A two-tailed, two-sided *t* test was used for all statistical analyses.

We found that the preTCR complex was constitutively removed from the cell surface at a significantly faster rate when compared with the αβTCR (Fig. 4 B). We hypothesized that the different internalization rates would also be observed in the intracellular routing of the two receptors once inside the cell. To specifically follow the fate of internalized receptors, we used receptor complexes with GFP fused to the extracellular terminus of TCRβ, as used for the RUSH assay, but without the SBP tag (Fig. 5 A). We could then incubate HEK cells expressing the receptors with the anti-GFP Nb to specifically label intracellular vesicles containing receptors that must have been endocytosed from the cell surface since the Nb cannot enter the cell unless directly bound to a receptor (Fig. 5 A). We then used live cell spinning-disk confocal microscopy to image the internalized Nb-bound receptors.

Within a few minutes of adding a fluorophore-conjugated anti-GFP Nb, labeled intracellular vesicles could be readily observed and these continued to accumulate with time (Fig. 5 B and Videos 3, 4, 5, and 6). To identify the type of vesicles that were Nb-positive, HEK cells expressing either αβTCR or preTCR were cotransfected with markers for either early (RAB5A) or late (RAB7A) endosomes along with a lysosomal marker (LAMP1; Fig. 5 A). We quantified the fraction of GFP-Nb positive vesicles that colocalized with these endosomes over a period of 2 h (Fig. 5, C and D). Both the internalized preTCR and TCR complexes colocalized with early endosomes (RAB5A^+^LAMP1^−^) within 5 min, but this pool decreased more rapidly for the preTCR (Fig. 5, C and D). In concordance with this, the preTCR was also present in the late endosome vesicle pool (RAB7A^+^LAMP1^−^) at the earlier time points and at a significantly higher fraction compared with the αβTCR (Fig. 5, C and D). Vesicles containing internalized preTCR showed a clear time-dependent transition to the lysosomal compartment (LAMP1^+^) that was very significantly different from the trafficking of the αβTCR complex (Fig. 5, C and D). Indeed, almost 60% of the preTCR vesicles were colocalized with lysosomes within 2 h (Fig. 5 C). The αβTCR vesicles remained colocalized with early endosomes for much longer, with minimal colocalization with late endosomes, and suggested the receptor was being recycled to the cell surface (Fig. 5, C and D). To confirm that the αβTCR complex was indeed being returned to the cell surface, we repeated the colocalization assay using a RAB11A marker that identifies recycling endosomes (Welz et al., 2014). As anticipated, a large fraction of internalized αβTCR vesicles colocalized with RAB11A, which implied the receptors were being recycled (Fig. 5, E and F; and Video 7). This colocalization was rarely observed for the preTCR (Fig. 5 F), however, reinforcing the point that when this receptor is endocytosed, it is routed directly for lysosomal degradation.

**Video 3.** **Internalized αβTCR colocalization with RAB5A in transfected HEK cells.** Video shows a representative HEK cell transiently expressing GFP-fused αβTCR complex in conjunction with mScarlet-RAB5A and LAMP1-BFP. A fluorescently labeled anti-GFP nanobody (Nb) is incubated with cells at t = 0 min, and the colocalization of internalized Nb with RAB5A vesicles quantified over time. Colored boxes denote protein representation in the overlaid images. Scale bar, 10 μm. Images were collected at 1 frames/s, with a playback rate of 5 frames/s.

**Video 4.** **Internalized αβTCR colocalization with RAB7A in transfected HEK cells.** Video shows a representative HEK cell transiently expressing GFP-fused αβTCR complex in conjunction with mScarlet-RAB7A and LAMP1-BFP. A fluorescently labeled anti-GFP nanobody (Nb) is incubated with cells at t = 0 min, and the colocalization of internalized Nb with RAB7A vesicles is quantified over time. Colored boxes denote protein representation in the overlaid images. Scale bar, 10 μm. Images were collected at 1 frames/s, with a playback rate of 5 frames/s.

**Video 5.** **Internalized preTCR colocalization with RAB5A in transfected HEK cells.** Video shows a representative HEK cell transiently expressing GFP-fused preTCR complex in conjunction with mScarlet-RAB5A and LAMP1-BFP. A fluorescently labeled anti-GFP nanobody (Nb) is incubated with cells at t = 0 min, and the colocalization of internalized Nb with RAB5A vesicles quantified over time. Colored boxes denote protein representation in the overlaid images. Scale bar, 10 μm. Images were collected at 1 frames/s, with a playback rate of 5 frames/s.

**Video 6.** **Internalized preTCR colocalization with RAB7A in transfected HEK cells.** The video shows a representative HEK cell transiently expressing the GFP-fused preTCR complex in conjunction with mScarlet-RAB7A and LAMP1-BFP. A fluorescently labeled anti-GFP nanobody (Nb) is incubated with cells at t = 0 min, and the colocalization of internalized Nb with RAB7A vesicles quantified over time. Colored boxes denote protein representation in the overlaid images. Scale bar, 10 μm. Images were collected at 1 frames/s, with a playback rate of 5 frames/s.

**Video 7.** **Internalized αβTCR colocalization with RAB11A in transfected HEK cells.** The video shows a representative HEK cell transiently expressing GFP-fused αβTCR complex in conjunction with mScarlet-RAB11A. A fluorescently labeled anti-GFP nanobody (Nb) was incubated with cells 10 min prior to imaging before simultaneous imaging of the Nb and RAB11A channels was acquired over ∼10 s. The first 20 frames of a representative movie are shown, with the colocalized movement of internalized Nb with RAB11A vesicles over time denoted with arrows. Colored boxes denote protein representation in the overlaid images. Video is related to Fig. 5 E of main text. Scale bar, 5 μm. Images were collected at 14.7 frames/s, with a playback rate of 5 frames/s.

In summary, these datasets point to the preTCR being internalized from the cell surface and efficiently trafficked through the endosomal compartments to lysosomes. This is in contrast to the αβTCR, which remains within the early/recycling endosomal fraction.

### Intracellular sequences of preTCR do not influence its cellular trafficking

**Figure 6.**
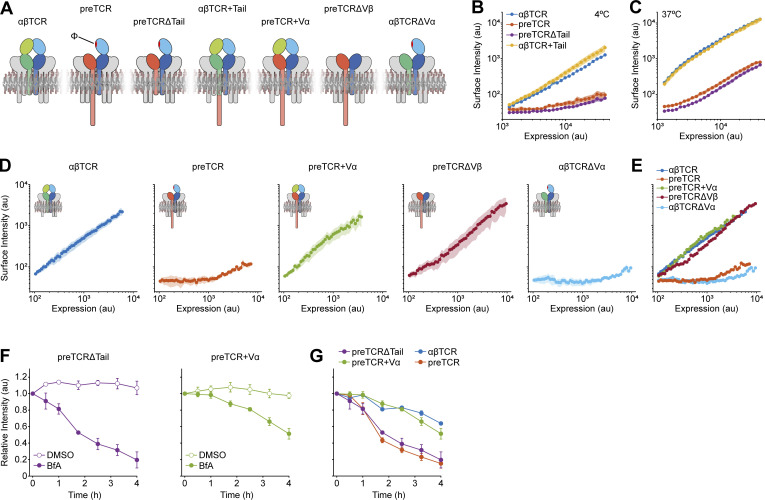
**Exposed extracellular preTCR domain drives constitutive internalization. (A)** Schematic showing the various receptor variants used in the figure. The exposed hydrophobic region (Φ) is marked in red. The intracellular region of CD3ζ chain has been removed for clarity but was present in all experiments. **(B)** Surface staining for TCRβ of receptors indicated in legend as a function of expression, with staining performed at 4°C to inhibit internalization. The intracellular sequence of preTCR (“Tail”) has no effect on constitutive surface expression. Data is shown as bounding area around datapoints with mean ± SEM (*n* = 3). **(C)** Equivalent datasets as in B but receptor-transfected HEK cells were incubated with anti-TCR antibody at 37°C for 30 min to identify any effect of the preTCR tail on the rate of internalization. Data is shown as a bounding area around data points with mean ± SEM (*n* = 3). **(D)** Surface staining for TCRβ of receptors indicated as a function of expression, with staining performed at 4°C to inhibit internalization. Data are shown as bounding area around datapoints with mean ± SEM (*n* = 3). **(E)** All datasets from D overlaid for direct comparison. **(F)** Brefeldin A (BfA) assay to measure the internalization rate of the indicated receptor variant, with vehicle control shown with open circles. Datapoints show mean ± SEM (*n* = 3). **(G)** Datasets from F overlaid with previous BfA assay datasets for αβTCR and preTCR complexes, which were collected at the same time to allow direct comparison. Datapoints show mean ± SEM (*n* = 3).

Thus far, we have shown that the preTCR has a phenotype of low surface expression caused by poor complex assembly in the ER and rapid removal from the cell surface when compared to the almost equivalent αβTCR. What feature of the preTCR then drives the trafficking within cells that we have observed? Previous reports have suggested that the long intracellular tail sequence of the pTa chain, not present in TCRα, controls the ER retention of the preTCR complex (Carrasco et al., 2001, 2003). This differentiating feature was used to explain the contrast between preTCR and αβTCR signaling in thymocytes. We were keen to see whether this potential retention motif within the preTCR could also explain our results with the reconstituted HEK cells. Thus, we made a new construct, preTCRΔTail, where the intracellular sequence of pTa was completely removed (Fig. 6 A), with the expectation that the preTCR should now readily traffic to the cell surface. However, we observed no substantial difference between the preTCR and preTCRΔTail in surface expression (Fig. 6 B). Equivalently, appending the intracellular sequence of pTa to TCRα, creating αβTCR+Tail (Fig. 6 A), did not impede the expression of the αβTCR at the cell surface (Fig. 6 B). We could also not observe any difference in internalization rate for these variants when incubated with a fluorescently labeled anti-TCRβ antibody at 37°C either (Fig. 6 C). Furthermore, repeating the BfA assay to quantify the receptor internalization rate showed no significant difference between the preTCR and preTCRΔTail (Fig. 6, F and G).

In combination, we find that it is not the unique intracellular sequence of the preTCR that explains the differential trafficking of this receptor when compared with the αβTCR.

### Extracellular structure of preTCR explains its divergent trafficking

If the trafficking of the preTCR cannot be ascribed to the intracellular region of the receptor complex, then perhaps the extracellular structure of the receptor could explain our results? The unique Ig domain of pTa could be directly responsible for directing receptor trafficking. An alternative explanation is that the exposed hydrophobic region (Φ) on TCRβ that is normally buried when interacting with TCRα might provide a unique recognition signal of the preTCR (Fig. 6 A). Thus, we engineered additional variants of the two receptors to directly explore these possibilities (Fig. 6 A). We reasoned that if the exposed hydrophobic surface was causing the rapid internalization of the preTCR, then fusing the variable domain of TCRα to the N-terminus of pTa (preTCR+Vα) would shield this region of TCRβ through the cognate interaction (Fig. 6 A). Expression of preTCR+Vα in HEK cells led to surface antibody staining that was now essentially indistinguishable from the αβTCR (Fig. 6, D and E). The addition of the Vα domain had completely abrogated the poor surface expression of the preTCR complex; would it also modulate its internalization? We repeated the BfA assay with the preTCR+Vα construct and found that it now phenocopied the αβTCR with sustained retention at the cell surface (Fig. 6, F and G).

**Figure S5.**
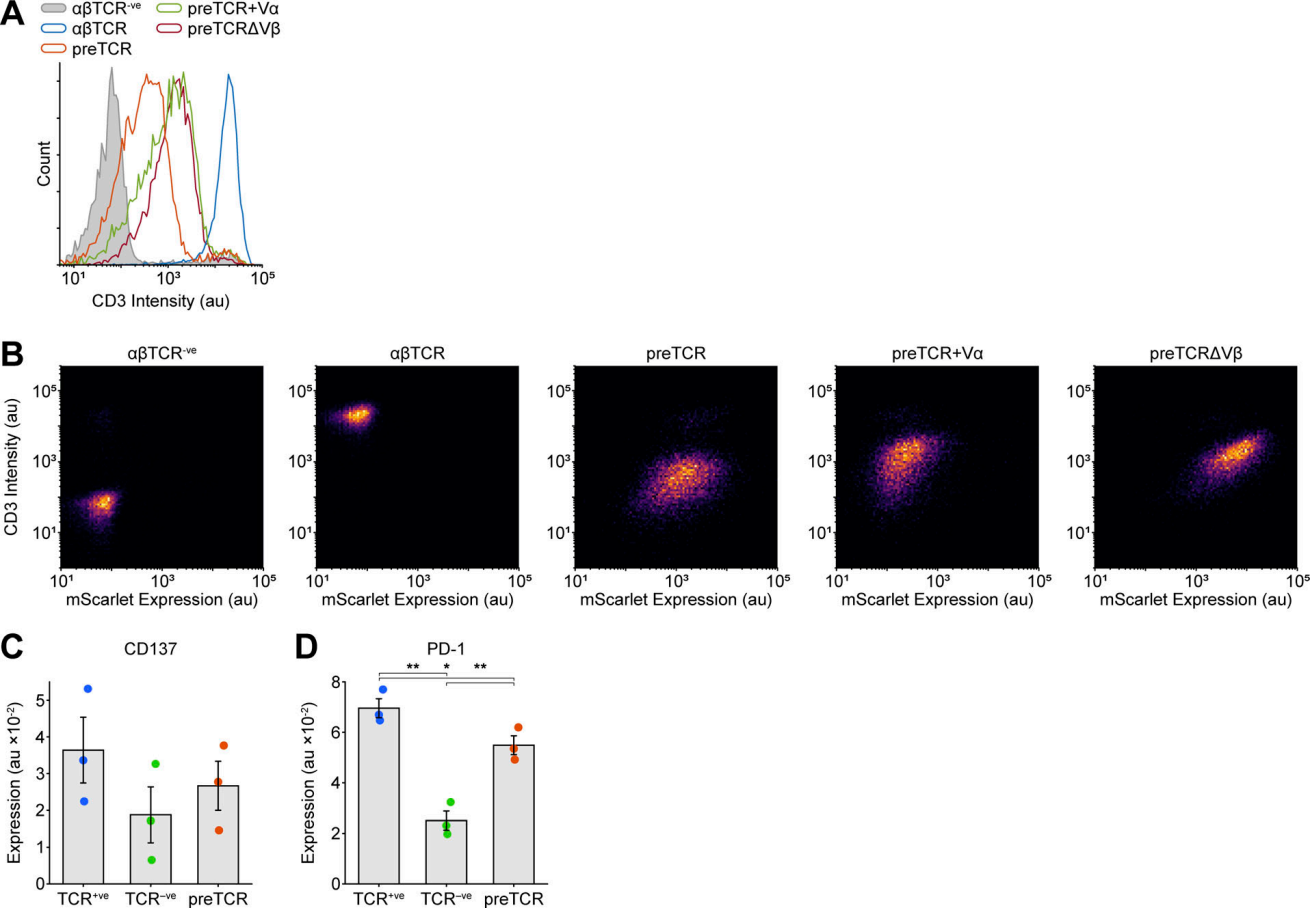
**Masking exposed hydrophobic region of TCRβ in preTCR increases surface expression in T cell line. (A)** TCR-negative Jurkats (αβTCR^−ve^) were lentivirally transduced with the indicated preTCR construct described in main text, and receptor surface expression, as measured by CD3 staining, was compared to wildtype Jurkat T cells (αβTCR). **(B)** Equivalent data as in A but now showing receptor surface expression levels as a function of preTCR variant expression (mScarlet fluorophore). **(C)** Quantification of CD137 expression on the three Jurkat T cell variants shown in Fig. 8. Error bars show mean ± SEM (*n* = 3). **(D)** Equivalent quantification as in C for PD1 expression on the three Jurkat variants. Error bars show mean ± SEM (*n* = 3); asterisks indicate P < 0.05 (*) or P < 0.01 (**) when comparing indicated datasets. A two-tailed, two-sided *t* test was used for all statistical analyses.

The preTCR+Vα construct includes the full sequence of the pTa chain; this point makes it implausible to explain our prior results on the preTCR to the only unique chain of the complex. Rather, it is the absence of the TCRα variable domain that is the explanation for the observed preTCR trafficking dynamics. To extend this hypothesis further, we removed the Vβ domain entirely from the preTCR (preTCRΔVβ) as an alternative means to abrogate exposure of the hydrophobic surface (Fig. 6 A). Concordant with our previous constructs, preTCRΔVβ was also well expressed at the cell surface (Fig. 6, D and E), reinforcing our conclusion that the unpaired Vβ domain exposing the hydrophobic region is the defining driver of preTCR trafficking dynamics. To consolidate this point, expression of either the preTCR+Vα or preTCRΔVβ construct in a TCR-negative T-cell line (described below) caused receptor surface expression at levels substantially greater than for the equivalent preTCR construct (Fig. S5, A and B).

Finally, we removed the Vα domain from αβTCR (αβTCRΔVα) to see if the receptor would now behave more like the preTCR due to exposing the normally buried surface of the Vβ domain (Fig. 6 A). This was indeed the observed result; transfection of αβTCRΔVα led to very poor surface expression when compared with the αβTCR complex (Fig. 6, D and E).

In summary, we have provided compelling evidence that it is the absence of a Vα domain in the preTCR complex that manifests its divergent trafficking compared with αβTCR (and potentially γδTCR) and not any part of the pTa protein sequence per se.

### The preTCR complex is monovalent at the cell surface

**Figure 7.**
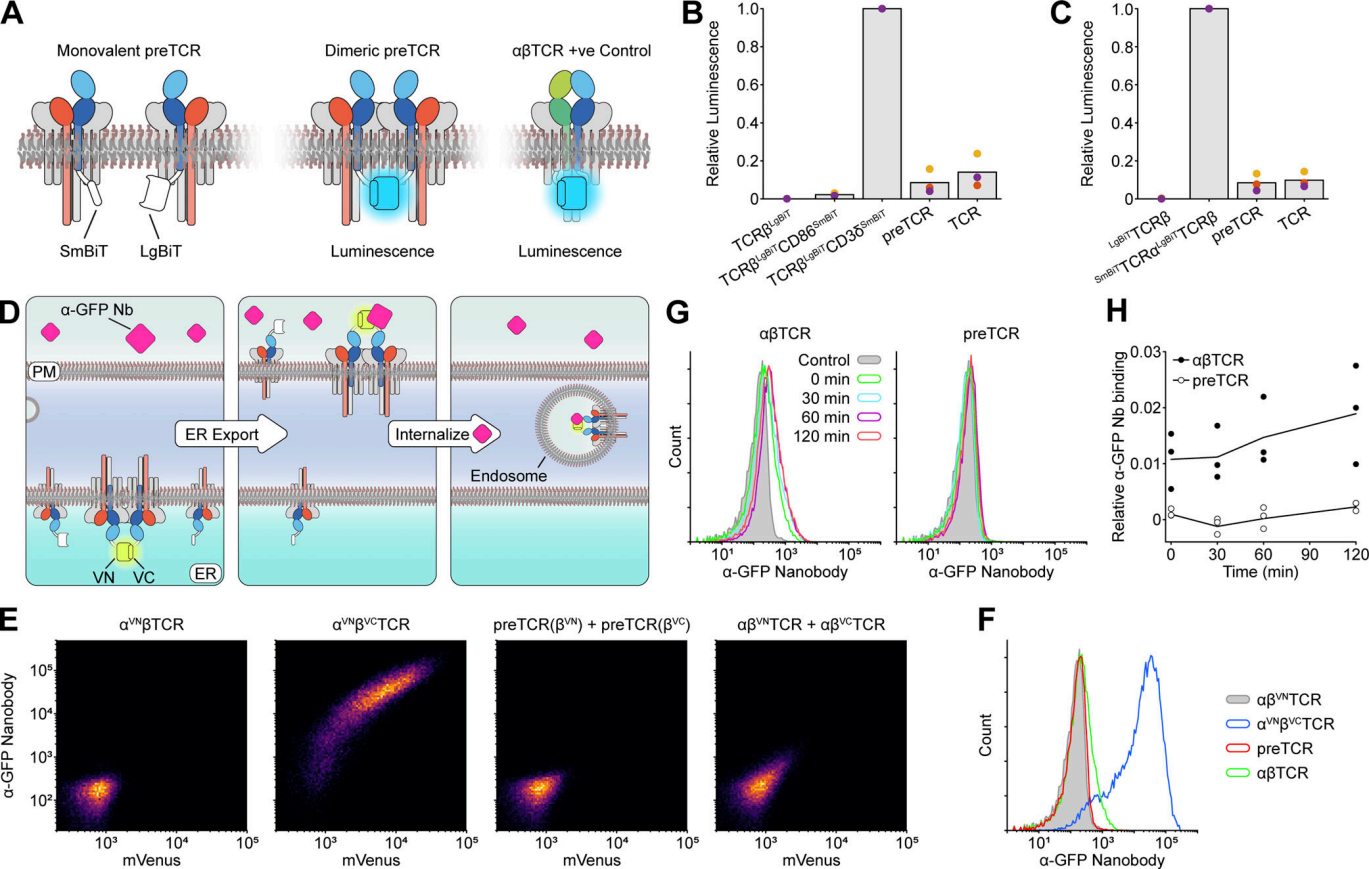
**preTCR complex shows no evidence for oligomerization at the cell surface. (A)** Schematic showing the fusion of the NanoBiT components to the intracellular sequences of the preTCR complex, together with αβTCR positive control constructs. **(B)** Luminescence from NanoBiT assay of indicated receptor constructs and controls, relative to the value for the αβTCR positive control. Datapoints for each of the three biological replicates are shown. **(C)** Luminescence from NanoBiT assay with component BiTs now localized to the extracellular side of the indicated receptors. Values are shown relative to the αβTCR positive control, with data points for each of three biological replicates shown. **(D)** Schematic showing the trimolecular fluorescence complementation assay, assuming the preTCR complex is multivalent. Reformation of the split Venus fluorophore through receptor dimerization affords labeled nanobody binding at cell surface, which can be internalized with time and detected by flow cytometry. **(E)** Flow cytometry plots showing the relationship between the reformed Venus fluorophore and nanobody binding. Positive control (α ^VN^β^VC^TCR) shows potent mVenus expression and concomitant nanobody binding, which is entirely absent from the preTCR sample. **(F)** Histogram showing nanobody binding at constitutive expression levels. **(G)** Representative datasets of nanobody binding to transfected HEK cells at 37°C with time, to allow potential internalization of undetectable receptor oligomers to be amplified. **(H)** Three biological replicates of datasets in G are quantified, with nanobody (Nb) staining shown with time and relative to the value measured for positive control.

Previous structural data on the extracellular domains of the preTCR (without CD3 chains) have suggested that the receptor dimerizes through complementary binding of the Vβ domain, which is the same region we find that induces low preTCR surface expression (Pang et al., 2010). Dimerization of the preTCR could therefore provide a mechanistic explanation for the rapid internalization we observe. However, the valency of the preTCR complex at the cell surface has not been directly investigated. Thus, we wanted to use our reconstituted system to investigate this question directly. We employed the NanoBiT assay, which is a very sensitive luminescence-based method to detect any potential oligomerization. In NanoBiT, two fragments of the NanoLuc luciferase (LgBiT and SmBiT) are genetically fused to the proteins of interest, which can associate to reform NanoLuc and emit detectable blue light if brought in sufficiently close proximity (Fig. 7 A).

We reasoned that by fusing the LgBiT or SmBiT to the intracellular terminus of TCRβ we would detect any potential preTCR dimers since this complex would bring TCRβ into close proximity (Fig. 7 A). We first confirmed that the assay was functional by appending LgBiT to TCRβ and SmBiT to CD3δ, two proteins known to associate within the αβTCR (and preTCR) complex (Fig. 7 A). Expression of the complete αβTCR complex with the modified TCRβ and CD3δ in HEK cells led to a readily detectable luminescence signal, confirming that the NanoBiT assay was functional for the TCR complex (Fig. 7 B). We also confirmed that LgBiT in isolation was neither capable of generating any signal above background nor nonspecific interactions with another membrane protein (CD86^SmBiT^; Fig. 7 B). When the assay was applied to preTCR dimerization, a luminescence signal was barely detectable and was lower than the equivalent signal observed for the αβTCR complex (Fig. 7 B). This result suggested that the preTCR has no more propensity to dimerize than the αβTCR. It was possible that the orientation of any potential preTCR dimerization precluded the association of the NanoBiT subunits fused to the intracellular end of TCRβ. Thus, we instead fused LgBiT or SmBiT to the cleaved N-terminus of TCRβ to detect the extracellular domains interacting within a potential preTCR complex dimer. Again, we found no evidence for significant preTCR complex dimerization within live cells (Fig. 7 C).

To measure the potential oligomerization of only plasma membrane–localized preTCR, we designed a trimolecular fluorescence complementation (TriFC) assay. The extracellular region of TCRβ was fused to either the N-terminal (VN) or C-terminal (VC) parts of split-Venus, where the fluorescence from this fluorescent protein can be detected when VN and VC are brought into close proximity (Fig. 7 D). To isolate only the cell surface pool of reconstituted mVenus, we incubated cells expressing these labeled receptors with the fluorescently labeled anti-GFP Nb used in earlier assays, which here can only bind to cells and be internalized if reformed mVenus is at the extracellular surface. A positive control of the αβTCR complex with VN-TCRα and VC-TCRβ confirmed that the assay worked, with mVenus fluorescence and Nb binding tightly correlated (Fig. 7, E and F). Expression of VN-TCRα tagged αβTCR alone caused no detectable mVenus fluorescence or Nb binding (Fig. 7, E and F).

Performing the assay with the preTCR led to undetectable levels of mVenus complementation or Nb binding (Fig. 7, E and F), while for the αβTCR, there was a very slight signal observed (Fig. 7, E and F). We also incubated cells expressing the TriFC constructs with the nanobody at different timepoints over 2 h to allow the gradual accumulation of fluorescence within cells to enhance any potential signal (Fig. 7, G and H). While for the TCR there was a subtle time-dependent increase in fluorescence, for the preTCR, there was essentially no accumulation of signal (Fig. 7, G and H).

In summary, we find no evidence for the association of preTCR complexes at the cell surface, which would negate dimerization as a mechanism for the rapid internalization of the receptor complex. These datasets also imply that dimerization is very unlikely to drive ligand-independent signaling of the preTCR. They further highlight that at the cell surface and likely within endosomal vesicles, the Vβ domain of the preTCR complex remains exposed.

### preTCR expression drives limited tonic signaling

There is no doubt that the preTCR must transmit a signal to the developing thymocyte to indicate that the β-selection checkpoint has been successfully crossed. If ligand binding is not an absolute requirement for preTCR function (Mahtani-Patching et al., 2011; Gibbons et al., 2001) and the receptor is monovalent, how then can a signal be generated simply by its transient presence at the cell surface? The preTCR, like the αβTCR, has no intrinsic enzymatic activity, and so relies on SRC-family kinases such as LCK to phosphorylate the tyrosine residues with its signaling motifs (ITAMs). This phosphorylation can be initiated and persists in regions of the cell surface where competing phosphatase activity is physically excluded (James and Vale, 2012; Chang et al., 2016). Pertinently, the αβTCR is also known to participate in tonic signaling, which is a minimal but constitutive signaling in T cells in the absence of cognate ligand binding (Myers et al., 2017).

**Figure 8.**
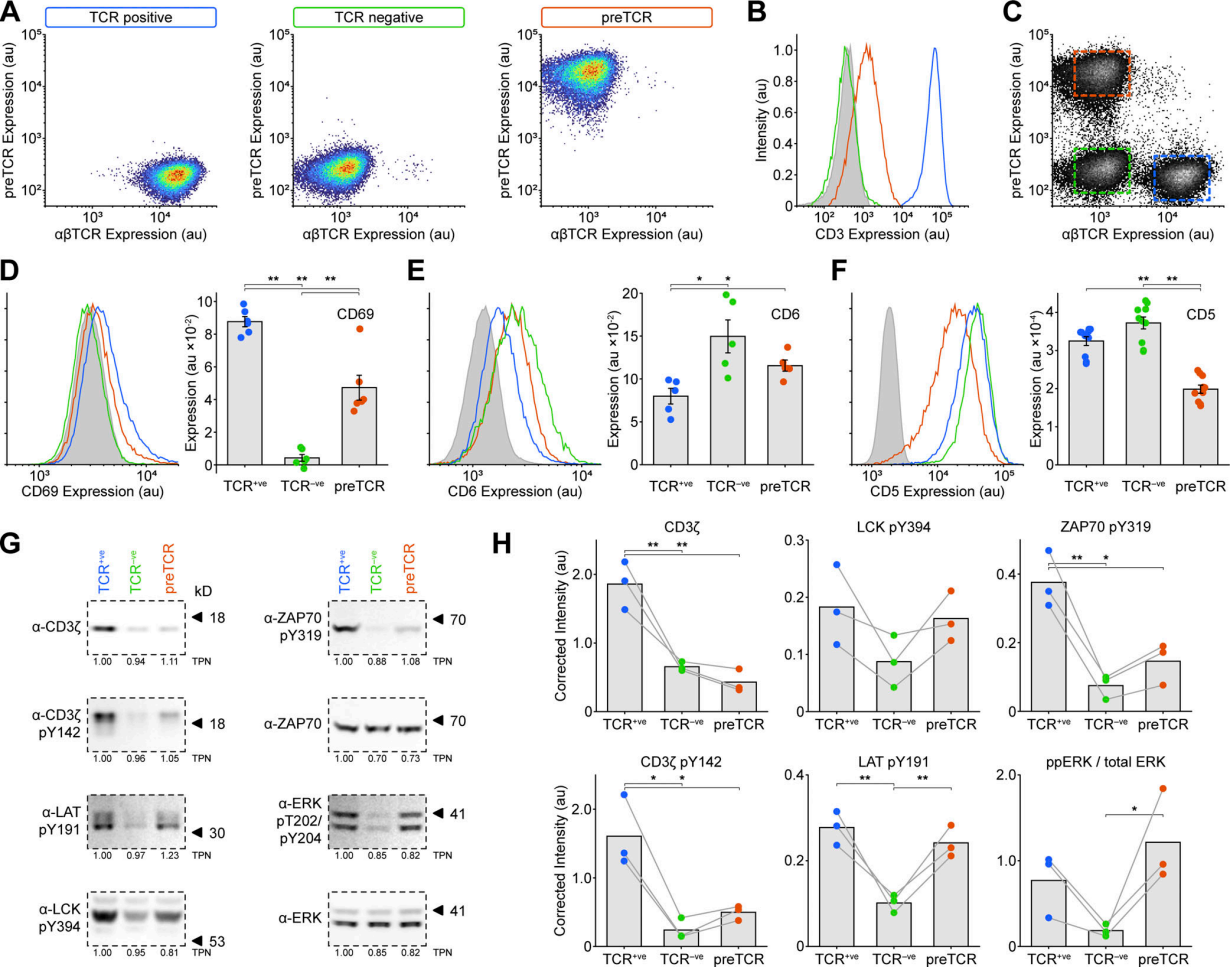
**preTCR expression can drive tonic signaling in a T cell line. (A)** Expression of the αβTCR (anti-αβTCR Ab) and preTCR (mScarlet) complexes on Jurkat T cells (“TCR positive”), Jurkats subjected to CRISPR/Cas9-mediated disruption of the *TCRA* gene (“TCR negative”) and these cells transduced with pTa-mScarlet to drive preTCR expression (preTCR). Boxes denote the color used throughout the figure for each cell line. **(B)** Surface staining for CD3 ε was used to confirm preTCR expression. TCR^+ve^ Jurkat cells (blue) show intense CD3ε staining that is completely lost on TCR^−ve^ cells (green) when compared to isotype control (filled grey). Expression of pTa-mScarlet (red) drives CD3ε surface staining demonstrating preTCR complex formation in these cells. **(C)** Flow cytometry plots showing all three cell lines could be easily separated when mixed in one experiment. **(D)** Left panel shows representative flow data for CD69 expression on the three Jurkat variants, colored as in B, and the right panel shows the quantification of CD69 expression. Error bars show mean ± SEM (*n* = 6); asterisks indicate P < 0.05 (*) or P < 0.01 (**) when comparing indicated datasets. **(E)** Equivalent datasets to D for CD6 expression. **(F)** Equivalent datasets to D for CD5 expression. **(G)** Representative Western images when blotting for denoted (phospho-)proteins for lysed samples of the three Jurkat variants shown in A. Relative total protein normalization (TPN) values under each blot correct for sample loading differences. **(H)** Quantification of Western images as shown in G, where each replicate is shown with connecting lines. Integrated band intensities were corrected for loading using TPN values. Bars show mean (*n* = 3). A two-tailed, two-sided *t* test was used for all statistical analyses. Source data are available for this figure: SourceData F8.

We hypothesized that the preTCR might also have the capacity for tonic signaling, which could provide a signal sufficient to cross the β-selection checkpoint. However, we could find no evidence to support or refute this idea. To provide a tractable system to test for tonic signaling, we made use of the Jurkat T cell line, which expresses αβTCR (Fig. 8 A; blue box). We used CRISPR/Cas9 to first ablate expression of TCRα from the rearranged *TCRA* locus in Jurkat cells, which led to undetectable αβTCR (Fig. 8 A; green box) or CD3ε expression at the cell surface (Fig. 8 B). We then expressed the *PTCRA* gene (fused to mScarlet fluorescent protein) to drive preTCR complex formation in Jurkats. A discrete population of αβTCR^−ve^, preTCR^+ve^ (preTCR) cells was isolated (Fig. 8 A; red box) that had detectable but very low (1.3% of αβTCR) CD3ε surface expression (Fig. 8 B), demonstrating that a functional preTCR complex was now trafficking to the plasma membrane.

To assess the relative levels of tonic signaling in these cells most accurately, all three populations were cultured together so that any differences between them must be an intrinsic property of their intracellular signaling and not any extracellular stimuli (Fig. 8 C). CD69 expression, a well-known activation marker, was essentially absent from the αβTCR-negative Jurkats (TCR^−ve^) but significantly upregulated in wildtype cells (TCR^+ve^), with the preTCR Jurkats expressing CD69 at an intermediate level (Fig. 8 D). Two other markers of activation, CD137 and PD-1, showed this trend, though at low-expression levels (Fig. S5, C and D). We also measured CD6 expression, which is thought to regulate the threshold of T cell development and function (Gonçalves et al., 2018; Oliveira et al., 2012; Orta-Mascaró et al., 2016) and is downmodulated on activation (Carrasco et al., 2017). CD6 showed the opposing trend to CD69, with increasing tonic signaling causing decreased CD6 expression (Fig. 8 E). Together, these datasets point to the preTCR providing a weak but detectable constitutive signal when expressed in a T-cell line. We were therefore intrigued when measuring the expression of CD5, a known marker of tonic signaling in thymocyte development (Myers et al., 2017; Azzam et al., 1998; Matson et al., 2020). The preTCR Jurkats had significantly reduced CD5 levels when compared with the other two populations (Fig. 8 F). Given preTCR expression was driving lower CD5 expression than cells with no receptor at all (TCR^−ve^), the preTCR might actively suppress CD5 gene expression, which is known to be low during early thymocyte development (Matson et al., 2020).

We also measured the phosphorylation state of several proteins involved in proximal TCR signaling in the three subsets (Fig. 8, G and H). As anticipated from the experiments above, expression of the CD3ζ chain subunit was most stable when bound within the αβTCR complex when compared with the TCR^−ve^ cells. CD3ζ chain expression was further decreased in preTCR Jurkats, likely due to active receptor degradation (Fig. 8, G and H). Despite this low level of expression, CD3ζ phosphorylation was readily detectable when compared with the TCR^−ve^ Jurkats. Phosphorylation of LCK (pY394), ZAP70 (pY319), and LAT (pY191) was increased for preTCR Jurkats when compared with TCR^−ve^ cells, reiterating that the expression of the preTCR alone can initiate weak but detectable tonic signals (Fig. 8, G and H). We also probed for ERK phosphorylation in the three cell lines, finding that this was more pronounced for the preTCR even than the αβTCR-expressing Jurkats.

In summary, we have shown that the preTCR is a receptor complex that is intrinsically poorly expressed and rapidly endocytosed, even in the absence of ligand binding. These distinctive features compared with αβTCR dynamics, and very likely the γδTCR, allow it to initiate weak but detectable tonic signaling in a manner that could be discriminated from stronger signaling from the TCR.

## Discussion

In this work, we have used a cellular reconstitution of the preTCR complex to provide mechanistic insight into how signaling from this developmentally important receptor might be distinguished from the γδTCR or aberrant αβTCR expression in thymocytes. We find that the asymmetric architecture of the preTCR extracellular domains drives both low surface expression and rapid endocytosis, which acts to blunt the duration of ligand-independent signaling from the receptor when compared with the αβTCR, and by extension potentially the γδTCR too. We also found no evidence for the intracellular sequence of the pTa protein controlling the trafficking of the preTCR. The strong concordance between the dynamics of the preTCR in HEK cells and thymocytes points to its characteristic functions determined by intrinsic factors.

The steady-state surface expression of a receptor is determined by the rate of its secretion to the cell surface as well as the rate at which it is removed from the cell surface by internalization and degradation. Our data suggest that the preTCR and αβTCR differ in both aspects. For a given expression level, more preTCR is retained in the ER than the αβTCR having failed complex assembly. Once assembled, however, the two complexes can traffic to the cell surface at comparable rates. When the preTCR is at the cell surface, it is rapidly internalized and directed toward lysosomal degradation, whilst the TCR is recycled back to the cell surface. It is important to note that these results were obtained through reconstituting receptor trafficking in non-immune cell lines in the absence of signaling kinases or other components of the TCR signaling cascade. That we can reconstitute the localization of the preTCR observed in thymocytes without the kinases suggests that receptor signaling, whether ligand-dependent or independent, is not a major determinant of receptor trafficking. This does not exclude the possibility that signaling may enhance preTCR turnover, as previously suggested (Panigada et al., 2002; Carrasco et al., 2001). In fact, both intrinsic and signaling-dependent internalization may synergize to ensure the efficient removal of the preTCR from the cell surface. However, given that signaling from the preTCR is thought to be weak, it would be far more consistent with the idea that its internalization would not be strictly dependent on signaling.

Contrary to previously published work, we find that the pTa cytoplasmic tail is not responsible for preTCR localization; we instead observe that the asymmetric extracellular structure of the receptor is responsible for rapid receptor internalization. There is very little evolutionary conservation in the intracellular pTa sequence, or its length, implying there are no consistent motifs present. An earlier study showed that transplanting this sequence to other receptors impeded their trafficking from the ER, but pertinently did not test this effect on the preTCR or αβTCR directly (Carrasco et al., 2001). It has been shown that the cytoplasmic tail, and specifically a proline-rich sequence, is necessary for the developmental progress of thymocytes passing the DN3 stage and into the DP stage (Borowski et al., 2004; Aifantis et al., 2002). However, other genetic studies in the mouse have shown that the pTa intracellular sequence is dispensable for thymocyte development, minimizing its role in β-selection (Gibbons et al., 2001; Fehling et al., 1997). Of note, these experiments were performed under non-competitive conditions that might mask a potentially poorer performance of these receptors.

We measured a significant difference in internalization rate between the αβTCR and preTCR, with the latter rapidly internalized via clathrin-mediated endocytosis, in agreement with observations made previously (Panigada et al., 2002; Carrasco et al., 2001). The stability of the αβTCR at the cell surface is not due to the lack of endocytosis, which has been shown to be continuously endocytosed and subsequently recycled in early endosomes and returned to the plasma membrane (Liu et al., 2000). It is therefore possible that the difference in cell surface stability between the αβTCR and the preTCR could be a combination of recycling of the former and retention in the endocytic compartment of the latter. This hypothesis is supported by the increase in surface preTCR complexes after incubation at 25°C, as previously found for “empty” MHC class-I molecules. Aside from being related, immune cell surface receptors, MHC class-I molecules, and the preTCR also have a similar structure. The extracellular domain of the empty class-I heavy chain has three Ig domains with an exposed hydrophobic patch at the interface between the heavy chain and β2 microglobulin; similarly, the preTCR has three extracellular Ig domains and an exposed hydrophobic patch on the side of the TCRβ chain variable domain that directly interacts with the variable domain of the TCRα chain. Because of these similarities, it is not unreasonable to expect that the same or a related mechanism may be behind the recognition and retention of internalized preTCR complexes.

Following this point, the question remains about how the structural information encoded in the extracellular domain architecture is transmitted across the plasma membrane. The simplest explanation would be that an unknown protein, possibly at the plasma membrane or within the early endosomes, specifically recognizes the extracellular structure of the preTCR. In analogy to the sorting of empty class-I heavy chains, the fate of endocytosed αβTCR and preTCR molecules from the plasma membrane would be determined within the early endosome. Despite our very considerable efforts to identify a potential chaperone or binding partner to direct the internalization of the preTCR, using proximity labeling assays such as PUP-IT (Liu et al., 2018), BioID2 (Kim et al., 2016), and APEX2 (Hung et al., 2016), no consistent protein could be found to explain the divergent routing of the preTCR compared with the αβTCR. Alternatively, the differential lipidation or other membrane interactions of the two receptor complexes may partition them into different parts of the endosome compartments that direct them to different fates.

We also posed an alternate hypothesis that preTCR oligomerization might explain its differential trafficking, but we could not find any evidence that the complete receptor complex clustered within cells to any extent greater than the αβTCR. Since the αβTCR is known to be monovalent (James et al., 2007, 2011; Brameshuber et al., 2018; Rossboth et al., 2018), this implied that the exposed hydrophobic surface of the TCRβ variable domain does not promote oligomerization. A previous study has suggested that preTCR oligomerization is mediated through the pTa domain (Yamasaki et al., 2006), but this study did not express the full receptor complex to probe stoichiometry, and the identified interaction would not be well-favored, based on recent structures of the complete αβTCR complex (Dong et al., 2019; Sušac et al., 2022). We believe that our direct interrogation of the preTCR complex provides the most direct evidence that, like the αβTCR, the preTCR is functionally monovalent.

Evidence has been published for an interaction between the preTCR and MHC molecules via the hydrophobic region on TCRβ (Mallis et al., 2015, 2018; Li et al., 2021). The genetic evidence that the β-selection checkpoint can be passed without the preTCR extracellular domains and also in MHC-I and MHC-II deficient mice would also indicate that if any interaction between the preTCR and MHC occurs in vivo then it is not an essential requirement for development (Mahtani-Patching et al., 2011). A recent study has shown that the absence of thymic MHC expression causes thymocytes to inefficiently commit to the double-positive (DP) stage, implying that MHC may more subtly enhance the signals received during β-selection (Duke-Cohan et al., 2022).

In summary, we have used a cellular reconstitution approach to investigate the trafficking dynamics of the preTCR complex in a far more tractable system and without the confounding effects of receptor signaling overlaid. We find that the preTCR is intrinsically poorly expressed at the plasma membrane and is far more transiently localized there when compared with the αβTCR. We identify the exposed hydrophobic surface of the TCRβ variable domain in the preTCR as the principal determinant of this effect. Finally, we present evidence that the preTCR complex is capable of tonic signaling in the absence of ligand binding, which we find is intermediate in intensity compared with tonic signals from the αβTCR and could provide sufficient “weak” stimulation to traverse the β-selection checkpoint.

## Materials and methods

### Reagents and resources

Key commercial and provided reagents that were critical to the present work are described in Table S1.

### Molecular cloning

The DNA sequences for all genes and constructs used in this study are provided in Table S2. All constructs were created by amplifying gene sequences using the polymerase chain reaction (PCR), incorporating appropriate restriction sites at the 5ʹ and 3ʹ ends of the sequence within the oligonucleotide primers. Unless described otherwise below, digested PCR products were ligated into the pHR-SIN lentiviral vector. In general, the various preTCR and αβTCR constructs were cloned into a bicistronic version of the pHR vector that uses the P2A self-cleaving protein sequence from Porcine Teschovirus-1 to stoichiometrically express two proteins (James, 2018). Where required, a gene encoding a fluorescent protein was inserted in-frame with the N- or C-terminus of the construct. Overlap extension (OE) PCR was used to genetically fuse different protein domains together when needed. When the components requiring joining were too small for OE PCR, the DNA sequence was synthesized as a gBlock (IDT) that could be directly amplified by PCR. For the RAB5A, RAB7A, RAB11A, and LAMP1 vectors, the genes were cloned into a pHR vector that has a weak promoter (mHSP) to drive the low level of expression. For the *B2M* gene knockout, CRISPR/Cas9 was used with oligonucleotides targeting sequences (5′-GGC​CGA​GAT​GTC​TCG​CTC​CG-3′ and 5′-GAG​TAG​CGC​GAG​CAC​AGC​T-3′) in the first exon of the *B2M* gene. For the *TCRA* gene knockout, CRISPR/Cas9 was used with oligonucleotides targeting a sequence (5′-GTT​GCA​CCT​CAG​CAG​AAC​CA-3′) in the *TRAC* exon of the rearranged *TCRA* gene. Chemically competent DH5α *Escherichia coli* were used to prep the DNA vectors, and the integrity of all amplified DNA sequences was confirmed by Sanger sequencing.

### Cell culture

HEK293T cells and HeLa cells were routinely cultured in T75 flasks at 37°C and 5% CO_2_ in DMEM supplemented with 10% heat-inactivated fetal bovine serum (HI-FBS), 2 mM *L*-glutamine, 100 U/ml penicillin, and 100 μg/ml streptomycin. Cells were grown to 90–100% confluency before passaging using 0.25% trypsin. The Jurkat T-cell line was routinely cultured in T25 flasks at 37°C and 5% CO_2_; in RPMI-1640 media supplemented with 10% HI-FBS, 2 mM *L*-glutamine, 100 U/ml penicillin, and 100 μg/ml streptomycin; and 1 mM HEPES buffer. Jurkat cells were grown between 0.1 and 1 × 10^6^ cells/ml and passaged by dilution in supplemented RPMI-1640.

### Cell transfection

HEK cells were seeded in 6-well plates at 5 × 10^5^ cells/well. After adhering overnight, GeneJuice (Merck) was used according to the manufacturer’s instructions to transfect 1–1.5 μg total DNA into each well. For all experiments, the total amount of DNA was held constant, using an irrelevant vector when needed to ensure this. Transfected cells were typically used 48 h after transfection for all the assays described. When stably expressing cell lines were required, lentiviral transduction was used essentially as described previously (James, 2018). Briefly, HEK cells were transiently transfected with 1.5 μg total DNA per well of a 6-well plate using the lentiviral backbone vector (derived from pHRSIN-CSGW), pCMVΔ8.91 (encoding essential HIV packaging genes), and pMD2.G (encoding VSV-G gene to pseudotype virus) at a 2:2:1 ratio. After 48–72 h, the supernatant was centrifuged at 14,000 × *g* for 2 min to remove debris and then incubated with ∼1 × 10^6^ target cells for 16 h. Fresh medium was then added, and the cells were recovered for at least 3 d. Expression of the transduced gene was monitored regularly by flow cytometry.

### Flow cytometry

For almost all samples to be analyzed by flow cytometry, cells were centrifuged at 800 × *g* for 3 min in standard 12 × 75 mm tubes and the pellet was resuspended in residual supernatant. When needed, primary antibodies were used at a working concentration of ∼10 μg/ml, diluted in flow wash buffer (2.0% [v/v] FBS, 0.1% [w/v], NaN_3_ in PBS, pH 7.4), and incubated on ice (except for internalization assays) for at least 20 min, with regular agitation. Samples were washed with 3 ml flow wash buffer and centrifuged as before, then fixed in flow fix buffer (1.6% [v/v] formaldehyde, 2% [w/v] glucose, 0.1% [w/v] NaN_3_ in PBS, pH 7.4). Either a BD LSRII or Fortessa, or Sony ID7000 spectral cytometer was used to acquire all data presented in this study. The flow cytometer was set up to ensure the most appropriate dynamic range between the negative and positive controls for each fluorescence channel, and at least 10,000 gated cells were collected for each sample. For analysis of flow cytometry datasets, the data were first gated based on scatter profiles in FlowJo or MATLAB, before further analysis in MATLAB to correct for the variable number of cells expressing the receptors at any given level, which is inherent to transient transfection. The data was first binned over a range of expression levels (x axis) and then the histogram of these binned values was created and normalized (y axis), which accounted for differences in absolute cell number within each bin. These normalized histograms at each binned expression level value are what is presented in the figures. When cells needed to be sorted, a BD Aria Fusion was used, with live cells collected in 12 × 75 mm tubes.

### Western blotting

When required, lysed samples were subjected to quantitative Western analysis. Unless stated otherwise, generally samples were prepared by mixing 80 μl resuspended cells with 30 μl 4× LDS buffer (Thermo Fisher Scientific) and 10 μl 1 M dithiothreitol. The samples were then incubated at 70°C for 10 min. A small volume (10–15 μl) of lysed samples was loaded onto precast 4–12% Bis-Tris NuPAGE gels (Thermo Fisher Scientific) and run at 200 V for 60 min using MES or MOPS running buffer (Thermo Fisher Scientific). Gels were then washed with deionized water and blotted onto nitrocellulose membranes using the iBlot2 apparatus (Thermo Fisher Scientific) according to the manufacturer’s instructions. The membranes were then incubated for 60 min in block solution (2.5% skimmed milk powder in TBS buffer, pH 7.5) before overnight incubation at 4°C with primary antibodies diluted in block solution supplemented with 0.1% Tween-20. Membranes were then washed three times for 5 min in TBS-T (0.1% Tween-20 in TBS) before incubation with secondary antibodies conjugated to AF647 or CF770 far-red/infrared dyes (1:1,000) for 60 min at room temperature. The membranes were subsequently washed three times for 5 min with TBS-T, once for 5 min with TBS, and then fluorescently imaged using the LI-COR Odyssey or Azure c600 imaging systems, according to the manufacturer’s instructions.

### Confocal microscopy

For routine live-cell imaging of transfected cells, we used a Nikon Ti inverted microscope equipped with a CSU-X1 spinning-disc confocal head (Yokogawa) and maintained at 37°C/5% CO_2_ with a thermally controlled enclosure (OKOLabs). Unless otherwise specified, a Plan Apo VC 100×/NA 1.4 oil-immersed objective (Nikon) was used for imaging onto an iXon Ultra EM-CCD camera (Andor) with a calculated pixel size of 134 nm, with 405, 488, 561, and 640 nm laser lines for fluorescent excitation. Fluorescence emission was collected through filters for mTagBFP (460 ± 15 nm), mGFP (525 ± 25 nm), mCherry/mScarlet (607 ± 18 nm), AF647/APC (685 ± 20 nm), and iRFP713 (708 ± 38 nm). The entire microscope system was controlled by μManager2 software that was used to create multichannel, multitime-point image data sets, and at multiple positions when required. The built-in perfect-focus unit of the microscope was used to correct axial focus drift due to fluctuations in temperature.

### Internalization assay

HEK cells were transiently transfected with either the preTCR or αβTCR complexes, where the C-terminus of TCRβ was fused to mGFP for quantification of receptor expression. 2 d after transfection, ∼1 × 10^6^ cells were harvested from the 6-well plate, washed with PBS, resuspended in 1 ml serum-free media, and prewarmed to 37°C. A 200-μl aliquot was first removed for measuring steady-state surface expression (on ice) before APC-conjugated αTCR Vβ5.1 (1:400) or AF647-conjugated αHA (1:200) antibodies were added to the media. The labeled cells were then incubated over a 2-h period at 37°C/5% CO_2_, with 200 μl aliquots taken at defined timepoints over the period and dispensed into tubes containing ice-cold flow wash buffer. Collected samples were then centrifuged at 800 × *g* for 3 min before fixing the resuspended pellet with flow fix buffer. Once all samples had been collected, they were acquired on a flow cytometer.

### BfA assay

HEK cells were transiently transfected in 6-well plates with plasmids encoding the CD3 chains and the required variant of the preTCR or αβTCR constructs. For the clathrin-mediated experiments, an additional plasmid encoding AP180C (Zhao et al., 2001) was also transfected. The following day, cells were harvested and the cell suspensions from two transfected wells were pooled. The samples were centrifuged at 800 × *g* for 3 min and the supernatant was discarded. The cell pellet was then resuspended in 1.5 ml serum-free medium and aliquoted into 14 100-μl samples, 7 for the drug-treated samples and 7 DMSO-treated samples in a 96-well plate. Separately, BfA (Sigma-Aldrich) or DMSO was added to 800 μl of serum-free media at a concentration of 2 μg/ml. 100 μl of BfA (or DMSO control) was added to wells at defined timepoints over 4 h. The plates were then centrifuged at 800 × *g* for 3 min and the supernatant was discarded. The plates were washed twice with 200 μl FACS Wash and pelleted as before. Cells were then incubated with AF647-conjugated α-HA antibodies diluted 1:100 in 50 μl FACS Wash and incubated for 30 min at 4°C. Plates were washed once with 150 μl FACS Wash and twice with 200 μl FACS Wash before fixation with 200 μl FACS Fix. The plates were then analyzed by flow cytometry using a cytometer equipped with a plate reader.

### Temperature-dependence assay

HEK cells were transiently transfected in 6-well plates with plasmids encoding the αβTCR or preTCR complexes. After 2 d, cells were removed from wells using trypsin and reseeded at low (∼50%) confluency in individual 35-mm dishes. After confirming cell adherence, dishes were moved to a 25°C/5% CO_2_ incubator overnight. Two control dishes were maintained at 37°C. The next day, each dish had the media changed (prewarmed to 37°C) and transferred to a 37°C/5% CO_2_ incubator for defined time periods. All cells were then harvested at the same time and stained with APC-conjugated α-TCR V_β_5.1 antibody for 30 min on ice. Cell surface receptor expression for each sample was then measured by flow cytometry.

### Glycosylation assay

HEK cells were transiently transfected with plasmids encoding the αβTCR or preTCR complexes, with the TCRβ chain tagged with eGFP at the C-terminus and an HA epitope at the cleaved N-terminus of TCRα or pTa, respectively. After 48 h, cells were trypsinized from plates and pipetted into 1.5-ml Eppendorf tubes. The samples were then centrifuged at 800 × *g* for 3 min at 4°C before washing cell pellets three times with 1 ml ice-cold PBS. After the final wash and removal of the supernatant, the cell pellet was mixed with 100 μl lysis buffer (1% NP-40 in Tris-buffered saline, supplemented with 100 mM NaV_i_, 200 mM PMSF, and 1× protease inhibitor cocktail [Life Technologies]) and incubated for 30–60 min on ice. The samples were then centrifuged at 11,000 × *g* for 10 min at 4°C. GFP-tagged TCRβ chains were affinity-purified using GFP-trap beads, as per the manufacturer’s instructions (Chromotek). The proteins were then eluted from the beads using 30 μl denaturation buffer (NEB) and split into three samples: 10 μl left untreated, 10 μl incubated with EndoH as per the manufacturer’s instructions (NEB), and 10 μl incubated with PNGase F, as per the manufacturer’s instructions (NEB). The final samples were then mixed with 10 μl 2× LDS loading buffer and Western blotted as described above. Western blots were stained with mouse α-HA antibodies diluted 1:1,000 and DyLight800-conjugated streptavidin diluted 1:10,000.

### CHX assay

To determine the relative resistance to degradation of the preTCR and αβTCR complexes, HEK cells were transiently transfected in 6-well plates to express the receptors additionally fused to mGFP for efficient comparison of expression. After 24 h, the cells in each well were treated with 100 μg/ml CHX (Sigma-Aldrich) overnight before they were harvested and lysed as described for the glycosylation assay. The protein abundance of the two receptors was analyzed by Western blotting, with blots stained with rabbit α-GFP antibodies (1:2,000) and rabbit α-actin subunit β (1:5,000). FIJI image analysis software was used to extract the band intensities within each lane of the Western blots, and MATLAB was used to quantify the integrated peak areas from these profiles.

### RUSH assay

HEK cells were seeded into 8-well microscopy dishes (ibidi) at a low density in 250 μl media. After 24 h, 4 wells were transiently transfected with the preTCR constructs, incorporating SBP–GFP fused to the cleaved N-terminus of TCRβ. These cells were also cotransfected with the ER hook (streptavidin-BFP-KDEL) and mCherry-CaaX to demark the plasma membrane. The other 4 wells were transfected with the corresponding set of constructs for the αβTCR. Cells were then imaged after 2 d using spinning disk confocal microscopy, maintained at 37°C/5% CO_2_, using a 40×/0.75 NA air objective. Nine positions were selected within each well allowing 4 wells, 2 expressing the preTCR and 2 expressing the TCR, to be imaged every 2 min. After the first frame, imaging was paused and 50 μl of GFP-Booster AF647 (Chromotek), 1:6,000 final dilution, was added to each well. After allowing 10 min (five frames) for the nanobody to diffuse throughout each well, 15 μl of biotin (Merck), 250 μM final concentration, was added to 2 of the 4 wells. Imaging proceeded for the next 90 min (45 frames). This procedure was then repeated for the other 4 wells. Repeating the assay but not including the SBP-tag on the receptors indicated that maximum nanobody binding occurred in 10 min in the absence of hook-mediated ER retention. Image stacks were subsequently analyzed in MATLAB. For each dataset, the number of fluorescent foci in the nanobody channel above a defined background was measured and results were averaged for each group. The Pearson correlation coefficient between the pixel intensity of the GFP channel (receptor) and intensities of the BFP (hook) and nanobody channels were also recorded for each frame.

### NanoBiT assay

Vector constructs were designed using SnapGene to fuse either the Large-BiT (LgBiT) or Small-BiT (SmBiT) of the NanoBiT assay to the C-termini of defined chains with the preTCR or αβTCR complexes. For confirmation of their expression, LgBiT and SmBiT were additionally tagged with mRuby2 and mTagBFP fluorophores, respectively. For the positive control with CD3δ fused to LgBiT, the intracellular sequence of CD3δ was truncated after the transmembrane to ensure that the two parts of the NanoBiT assay were equivalently distanced from the bilayer. The NanoBiT constructs were transiently transfected into HEK cells with defined LgBiT and SmBiT-fused receptor pairings. After 48 h, transfected cells were removed from 6-well plate, pelleted, and resuspended in 200 μl PBS before aliquoting 100 μl into 2 wells of a white 96-well plate (∼2.5 × 10^5^ cells/well). Luminescence (1 s integration time) was determined using the NanoGlo kit, according to the manufacturer’s instructions (Promega). Fluorescent emission from the fluorophores was also acquired for normalizing the luminescent signal to receptor expression. For the NanoBiT assay with the two subunits localized to the extracellular side of the receptors, LgBiT or SmBiT were fused to the mature N-terminus of TCRβ in the preTCR and αβTCR complexes. For confirmation of LgBiT or SmBiT expression, the C-terminus of TCRβ was tagged with mGFP or mScarlet fluorophores, respectively. The positive control construct was created by fusing SmBiT to the mature N-terminus of TCRα in the LgBiT-TCRβ construct. Otherwise, the assay was performed as described above. Luminescence and fluorescence values were measured using either a Tecan or Cytation5 plate reader.

### RAB colocalization assay

Constructs were designed that incorporated mGFP at the mature N-terminus of TCRβ in both the αβTCR and preTCR vectors. The red fluorescent protein (FP) mScarlet was fused to the N-terminus of RAB5A, RAB7A, or RAB11A and cloned into a vector that used a weak promoter to minimize their overexpression. Similarly, LAMP1 was fused to the blue FP mTagBFP and cloned into an equivalent vector. HEK cells were transfected with plasmids encoding (αβTCR/preTCR), (RAB5A/RAB7A/RAB11A), and LAMP1 at a 2:1:1 ratio in 6-well plates. The following day, transfected cells were trypsinized from wells and seeded at low density into fibronectin-treated 8-well microscope dishes (ibidi). After a further day for cells to adhere, cells were imaged by spinning-disk confocal microscopy at 37°C/5% CO_2_ as described above.

For the movies, cells confirmed to be expressing all components were identified and positions were marked before the addition of AF647-labeled anti-GFP Nb (1:2,000 final dilution) while taking images in all channels every 1 min over 40–60 min. For the colocalization datasets, cells were similarly transfected and replated. However, after anti-GFP Nb addition, a range of positions (9–13 per timepoint) was imaged at each defined time point over the course of 2 h. For quantifying the colocalization, the multichannel image at each time point was analyzed using MATLAB. A custom interactive script was used to first manually define the interior of the cell before automated filtering and thresholding of the anti-GFP Nb image into a binary mask. The centroids of connected pixels within the mask were defined and the intensity in each fluorescent channel at these points was manually scored for vesicular colocalization between the nanobody, RAB5A/RAB7A, and LAMP1 images. The fraction of nanobody-staining vesicles colocalized for each of these markers was then quantified for each cell, and this is what is presented in the figures. Overall time points and repeats, a total of ∼10,000 vesicles, were scored.

For the RAB11A datasets, it was not possible to image colocalization using sequential channel acquisition as the RAB11A vesicles moved too rapidly. Instead, an FV30-FRCY5 (Olympus) beam splitter was positioned in the TuCam unit (Andor) to allow simultaneous imaging of the mScarlet-RAB11A and AF647-labeled anti-GFP Nb datasets. A razor blade was used to align the filter cube to provide first-pass registration of the two camera images using TetraSpeck beads as the reference. For final image registration, an affine transform of the imaged TetraSpeck beads was calculated using MATLAB, which was then applied to the RAB11A datasets and provided excellent alignment of the two channels. The spillover of mScarlet fluorescence into the AF647 channel was calculated (∼15%) and corrected prior to image analysis. Vesicle colocalization was then scored as described above for RAB5A and RAB7A.

### Trimolecular fluorescence complementation assay

Following a similar cloning strategy to the extracellular NanoBiT assay, the VN or VC fragments of mVenus (Ohashi et al., 2012) were fused to the mature N-terminus of TCRβ in both the preTCR and αβTCR vectors. For the positive control, VN was fused to the N-terminus of TCRα within the αβ^VC^TCR vector. HEK cells were transiently transfected with the receptor complexes over 2 d before being stained on ice using the AF647-labeled anti-GFP Nb (1:1,000 final dilution), which is known to recognize mVenus due to their almost equivalent protein sequence (Croucher et al., 2016). The binding of the Nb to cells requires the VN and VC to have dimerized to reform the mVenus protein, hence Nb staining is directly reported on the stoichiometry of the receptors. Flow cytometry was used to quantify the intensity and correlation of the mVenus and anti-GFP Nb fluorescence. For measuring the time-dependent increase in Nb, internalization, transfected cells were replated into 24-well plates after 24 h. The following day, Nb was added to cells at defined time points before trypsinizing cells, washing, and resuspending in flow fix buffer. In this assay, the zero time point corresponded to transfected cells stained with the Nb on ice for 30 min.

### Data analysis

All data analysis was performed either with FIJI/ImageJ or MATLAB using custom scripts where necessary to extract information from the datasets. For all statistical tests, an unpaired, two-sided two-sample *t* test was used (assuming equal variance) to calculate P values presented in the main text. Data were assumed to be distributed normally but this was not formally tested.

### Online supplemental material

Fig. S1 shows preTCR internalization in the HeLa and Jurkat cell lines. Fig. S2 shows that the complete preTCR complex is present at the cell surface and it is not phosphorylated in HEK cells. Fig. S3 shows that preTCR surface expression can be increased by decreasing temperature. Fig. S4 shows that the presence of MHC expression on HEK cells does not affect observed preTCR internalization. Fig. S5 shows that masking the normally exposed hydrophobic extracellular region of preTCR increases its surface expression in a T cell line, and that preTCR can drive increased expression of CD137 and PD-1 through tonic signaling. Video 1 shows one RUSH assay dataset used to measure the trafficking of the αβTCR. Video 2 shows one RUSH assay dataset used to measure the trafficking of the preTCR. Video 3 shows colocalization of internalized Nb-bound αβTCR with RAB5A vesicles. Video 4 shows colocalization of internalized Nb-bound αβTCR with RAB7A vesicles. Video 5 shows colocalization of internalized Nb-bound preTCR with RAB5A vesicles. Video 6 shows colocalization of internalized Nb-bound preTCR with RAB7A vesicles. Video 7 shows colocalization of internalized Nb-bound αβTCR with RAB11A vesicles. Table S1 lists the key resources used in the study. Table S2 lists the DNA sequences of all constructs used in the study.

## Supplementary Material

Table S1 lists the key resources used in the study.

Table S2 lists the DNA sequences of all constructs used in the study.

SourceData F1 is the source file for Fig. 1.

SourceData F8 is the source file for Fig. 8.

SourceData FS2 is the source file for Fig. S2.

## Data Availability

The data are available from the corresponding author upon reasonable request.
