# Supplementary figures and images for: Pre-T cell receptor localization and trafficking are independent of its signaling
Source: J Cell Biol. 2023 Jul 26;222(10):e202212106. doi: 10.1083/jcb.202212106 (PMC10373305; doi:10.1083/jcb.202212106)

# SourceData F1

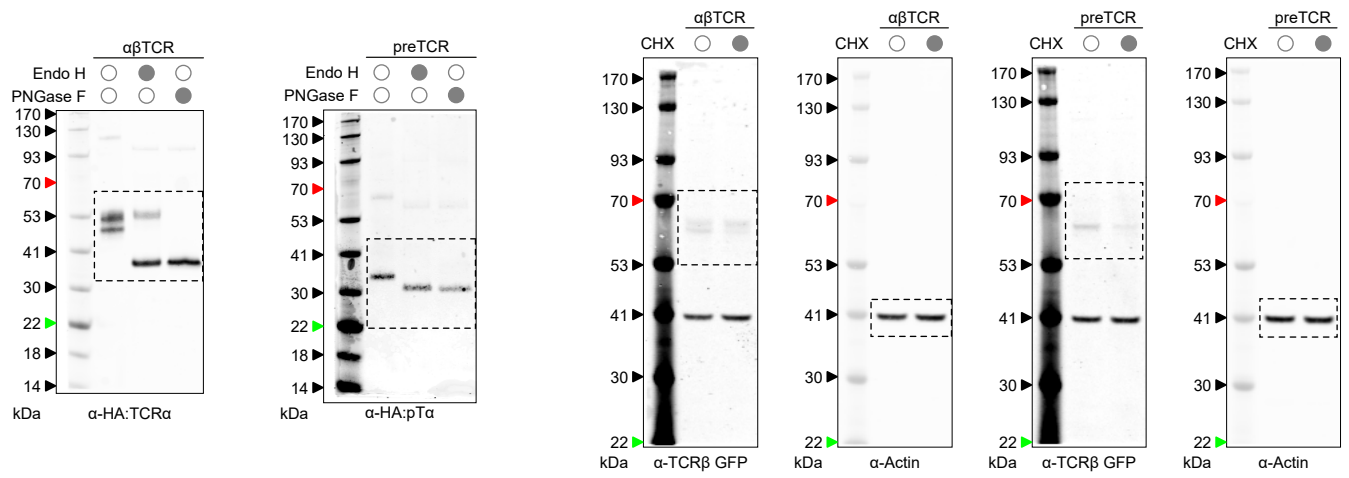

Supplement: SourceData F1 — is the source file for Fig. 1. [file JCB_202212106_SourceDataF1.pdf]

# SourceData F8

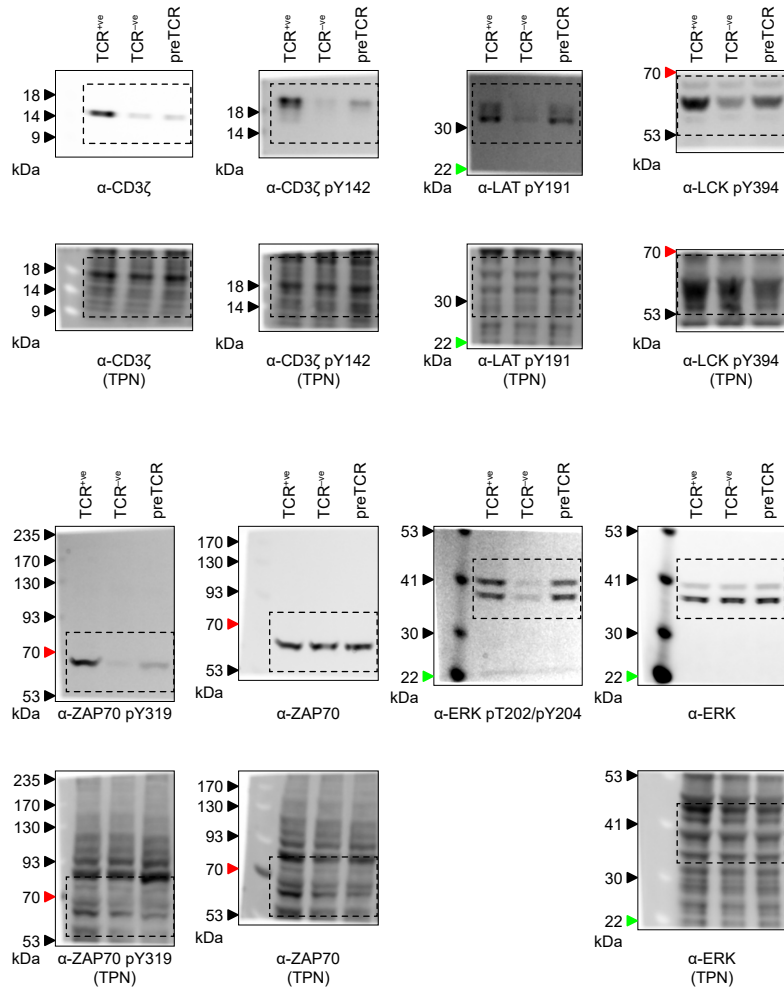

Supplement: SourceData F8 — is the source file for Fig. 8. [file JCB_202212106_SourceDataF8.pdf]

# SourceData FS2

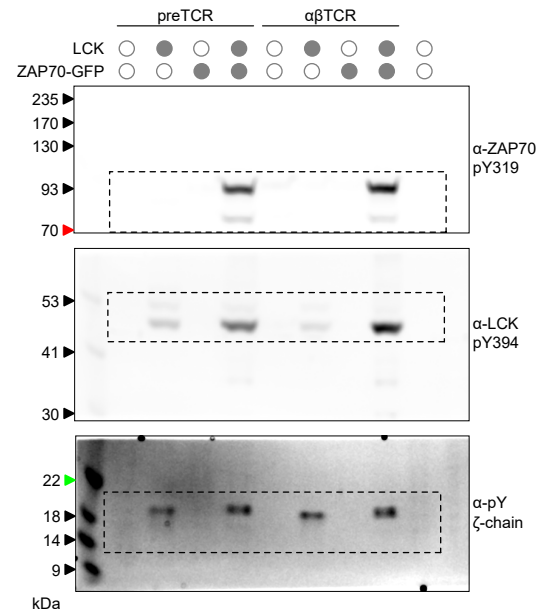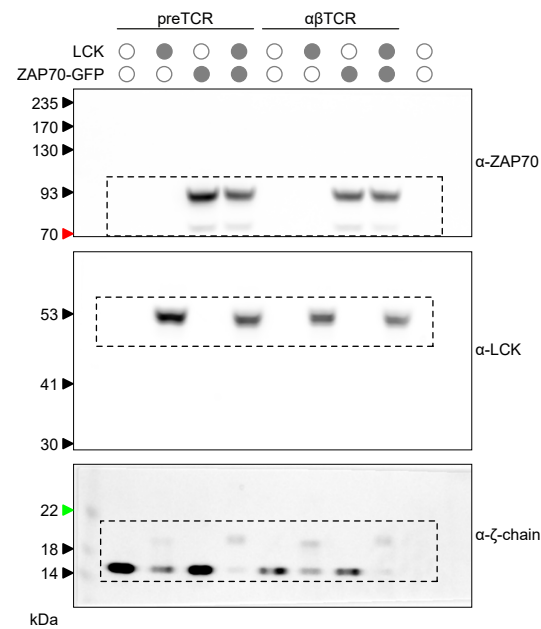

Supplement: SourceData FS2 — is the source file for Fig. S2. [file JCB_202212106_SourceDataFS2.pdf]
